# Sulfate-Reducing Bacteria That Produce Exopolymers Thrive in the Calcifying Zone of a Hypersaline Cyanobacterial Mat

**DOI:** 10.3389/fmicb.2019.00862

**Published:** 2019-04-24

**Authors:** Stefan Spring, Dimitry Y. Sorokin, Susanne Verbarg, Manfred Rohde, Tanja Woyke, Nikos C. Kyrpides

**Affiliations:** ^1^Department Microorganisms, Leibniz Institute DSMZ-German Collection of Microorganisms and Cell Cultures, Braunschweig, Germany; ^2^Winogradsky Institute of Microbiology, Research Centre of Biotechnology, Moscow, Russia; ^3^Department of Biotechnology, Delft University of Technology, Delft, Netherlands; ^4^Department Services Microorganisms, Leibniz Institute DSMZ-German Collection of Microorganisms and Cell Cultures, Braunschweig, Germany; ^5^Central Facility for Microscopy, Helmholtz Centre for Infection Research, Braunschweig, Germany; ^6^DOE Joint Genome Institute, Walnut Creek, CA, United States

**Keywords:** stromatolites, lithification, biofilm, alkalinity engine, sulfate reduction

## Abstract

Calcifying microbial mats in hypersaline environments are important model systems for the study of the earliest ecosystems on Earth that started to appear more than three billion years ago and have been preserved in the fossil record as laminated lithified structures known as stromatolites. It is believed that sulfate-reducing bacteria play a pivotal role in the lithification process by increasing the saturation index of calcium minerals within the mat. Strain L21-Syr-AB^T^ was isolated from anoxic samples of a several centimeters-thick microbialite-forming cyanobacterial mat of a hypersaline lake on the Kiritimati Atoll (Kiribati, Central Pacific). The novel isolate was assigned to the family *Desulfovibrionaceae* within the *Deltaproteobacteria*. Available 16S rRNA-based population surveys obtained from discrete layers of the mat indicate that the occurrence of a species-level clade represented by strain L21-Syr-AB^T^ is restricted to a specific layer of the suboxic zone, which is characterized by the presence of aragonitic spherulites. To elucidate a possible function of this sulfate-reducing bacterium in the mineral formation within the mat a comprehensive phenotypic characterization was combined with the results of a comparative genome analysis. Among the determined traits of strain L21-Syr-AB^T^, several features were identified that could play a role in the precipitation of calcium carbonate: (i) the potential deacetylation of polysaccharides and consumption of substrates such as lactate and sulfate could mobilize free calcium; (ii) under conditions that favor the utilization of formate and hydrogen, the alkalinity engine within the mat is stimulated, thereby increasing the availability of carbonate; (iii) the production of extracellular polysaccharides could provide nucleation sites for calcium mineralization. In addition, our data suggest the proposal of the novel species and genus *Desulfohalovibrio reitneri* represented by the type strain L21-Syr-AB^T^ (=DSM 26903^T^ = JCM 18662^T^).

## Introduction

Lithifying microbial mats represent probably the earliest ecosystems on Earth that started to appear more than three billion years ago and were preserved in the fossil record as stromatolites (Grotzinger and Knoll, 1999; Nutman et al., 2016). Calcification plays a main role in the lithification process of laminated microbial mats and can be explained by two main factors: an alkalinity engine within the mat and the properties of the organic mat matrix creating localized supersaturating conditions for calcium carbonate (Dupraz et al., 2009). The so-called alkalinity engine designates the potential of a microbial community to increase the local pH by its metabolic activity. An increase of pH can be induced mainly by photosynthesis or sulfate reduction and may promote mineralization by increasing the availability of carbonate. The organic mat matrix is composed of extracellular polymeric substances (EPS) produced by oxygenic photoautotrophic microorganisms thriving in the upper layers of the mat. Most functional groups of freshly produced EPS are acidic and have the property to bind bivalent cations, especially calcium. Degradation of EPS by heterotrophic bacteria in deeper mat layers can lead to the release of calcium thereby stimulating mineral precipitation.

The importance of sulfate reduction in the lithification of microbial mats has been postulated for a long time (e.g., Jørgensen and Cohen, 1977; Lyons et al., 1984; Visscher et al., 1998) and is mainly based on the following considerations: increase of the calcium carbonate saturation index (SI) during growth of sulfate-reducers with hydrogen or formate (Gallagher et al., 2012), reduction of the sulfate-dependent inhibition of carbonate precipitation (Wright and Wacey, 2005), release of calcium by the degradation of functional groups of EPS (Glunk et al., 2011), and provision of nucleation sites promoting mineral precipitation (Braissant et al., 2007). Indirect evidence for the involvement of sulfate reduction in the lithification process was presented in several studies in which the highest sulfate reduction rates were detected in zones of active calcium mineral precipitation (Visscher et al., 2000; Dupraz et al., 2004; Glunk et al., 2011; Pace et al., 2018).

Despite their assumed importance, only a few isolates of sulfate-reducing bacteria have been obtained from the lithification zones of microbial mats. Previously, the strains H12.1 and H2.3j were isolated from lithifying surface mats of Highborne Cay stromatolites and used in growth experiments to demonstrate the effect of EPS production and substrate utilization on calcium mineralization (Braissant et al., 2007; Gallagher et al., 2012). Unfortunately, no detailed descriptions of their phenotypes were provided, but based on the available partial 16S rRNA gene sequences (DQ822785, DQ822786) these strains are closely related to the widespread species *Halodesulfovibrio marinisediminis* (99% sequence identity). In addition, the strain LVform1 was retrieved from anoxic sediments of a shallow hypersaline coastal lagoon in Brazil (Warthmann et al., 2005). It represents the type strain of the species *Desulfovibrio brasiliensis* and was used in several studies demonstrating microbial influenced formation of dolomite (e.g., Bontognali et al., 2014). Only recently the isolation of a sulfate-reducing bacterium that is specifically associated with the transition zone of a lithifying, hypersaline microbial mat was achieved (Spring et al., 2015a). In this study, we report the phenotypic and genomic characteristics of the novel strain and correlate these traits with a possible function in the biogeochemistry of the studied microbial mat.

## Materials and Methods

### Strains and Cultivation Conditions

Strain L21-Syr-AB^T^ was isolated from an anaerobic enrichment culture with syringate as substrate, inoculated with slurries of a cyanobacterial mat sample retrieved from the hypersaline Lake 21 on the Kiritimati Atoll (Northern Line Islands, Republic of Kiribati). The location of the sampling site and details of the isolation procedure were described previously (Spring et al., 2015a). For the preparation of media and incubation under anoxic conditions the anaerobe cultivation technique of Hungate (1950) with the modifications introduced by Bryant (1972) was used. For routine cultivation of strain L21-Syr-AB^T^ the medium DSM 1526c was used that contained per liter deionized ultrapure water: 60.0 g NaCl, 6.0 g MgSO_4_ × 7 H_2_O, 1.5 g KCl, 1.0 g Na_2_S_2_O_3_ × 5 H_2_O, 1.0 g NH_4_Cl, 0.4 g CaCl_2_ × 2 H_2_O, 0.4 g K_2_HPO_4_, 10.0 ml trace elements solution of DSMZ medium 141^1^, 1.0 g yeast extract, 0.5 mg resazurin, 10.0 ml vitamins solution of DSMZ medium 141, 1.5 g Na_2_CO_3_, 2.5 g sodium pyruvate, and 0.5 g Na_2_S × 9 H_2_O. The medium was prepared under 80% N_2_ and 20% CO_2_ gas mixture without the vitamins, carbonate, sodium pyruvate and sulfide, which were added to the medium after autoclaving from sterile anoxic stock solutions. The pH of the completed medium was adjusted to 7.3 – 7.5 and the standard incubation temperature was 35°C. For testing carbon source utilization, sodium pyruvate was omitted and the amount of yeast extract was reduced to 0.05 g l^-1^. Fermentative growth was determined in a sulfate-free medium 1526c that was prepared without yeast extract and Na_2_S_2_O_3_ × 5 H_2_O. In addition, MgSO_4_ × 7 H_2_O was replaced with MgCl_2_ × 6 H_2_O and the trace elements solution of DSMZ medium 141 with the trace elements of DSMZ medium 320^2^. Pure chemicals were obtained from Sigma-Aldrich^3^ and complex nutrients from BD Biosciences ^4^.

For comparison, the type strains *Desulfovibrio alkalitolerans* DSM 16529^T^ and *Desulfovibrio africanus* subsp. *africanus* DSM 2603^T^ were obtained from the Leibniz Institute DSMZ. For cultivation of these strains the medium recipes and incubation conditions indicated in the microorganisms catalog of the Leibniz Institute DSMZ were used^5^.

### Analyses of Phylogeny and Spatial Distribution

Phylogenetic analyses were based on an alignment of 16S rRNA gene sequences included in the SILVA database SSU Ref NR 99 release 132 (Quast et al., 2013). Calculations were limited to a selection of sequences comprising type strains affiliated with the *Desulfovibrionales* supplemented with several representative environmental sequences of uncharacterized or uncultured bacteria having a recognizable relatedness to the strain L21-Syr-AB^T^. For complementary analyses, protein sequences from the respective *rpoB*, *dsrA* and *dsrB* genes were identified from the genome sequences obtained from NCBI GenBank or the DOE Joint Genome Institute (JGI) Integrated Microbial Genomes and Microbiomes (IMG/M) system (Chen et al., 2018). The specifications of genomes selected for the reconstruction of phylogenetic relationships are shown in Supplementary Table S1. Protein sequences were aligned using the ClustalW algorithm implemented in the ARB package (Ludwig et al., 2004). The sequences of DsrA and DsrB were concatenated prior to further calculations. Phylogenetic trees based on data sets of 16S rRNA gene or protein sequences were reconstructed using programs implemented in the ARB software package. When the ARB neighbor-joining program was used, phylogenetic distances were calculated with the corrections of Jukes-Cantor for nucleic acids and Kimura for proteins. Maximum likelihood trees were reconstructed using RaxML (version 7.7.2) with the GTRGAMMA model for DNA and PROTCAT-LG for proteins under the rapid bootstrap analysis algorithm. The maximum parsimony program of PHYLIP included in the ARB package was used with default settings for nucleotide (dnapars) or amino acid (protpars) sequences. The robustness of tree topologies was evaluated by performing 1000 rounds of bootstrap replicates. Identity values of gene sequences were determined for 16S rRNA genes using the similarity option of the ARB neighbor-joining program, while for nucleotide sequences of protein genes the pairwise alignment tool of the discontiguous megablast algorithm was used^6^.

The vertical distribution of various clades of sulfate-reducing bacteria within the mat from top to bottom was analyzed as described previously (Ben Hania et al., 2017). The reported relative abundance values are based on a data set of partial 16S rRNA gene sequences deposited under the NCBI Sequence Read Archive project accession number SRA058120 (Schneider et al., 2013). The proportion of sequences affiliated with a species-level clade represented by strain L21-Syr-AB^T^ was determined using a minimum sequence identity of 97% with the deposited 16S rRNA gene sequence of this isolate (KC665952). For the purpose of this study, we have refrained from using the more stringent species threshold of 98.7% (Yarza et al., 2014) because the amplicon sequences are only about 520 nucleotides in length, preventing accurate analysis.

### Characterization of the Phenotype

Cell shape, dimensions, spore formation and motility were examined by phase-contrast microscopy. Samples for scanning electron microscopy were prepared according to the protocols described in Wittmann et al. (2014). Gram-staining was carried out using the kit of Merck (Germany) according to the instructions of the manufacturer. Growth of strain L21-Syr-AB^T^ with different electron donors was determined in liquid medium by measuring the increase in optical density at 430 nm and the production of sulfide measured by the method of Cord-Ruwisch (1985). The end products of fermentation were analyzed by gas chromatography according to published protocols (Holdemann et al., 1977; Steer et al., 2001).

Presence of desulfoviridin was determined by the method of Postgate (1959). The dominant cytochrome types were identified in cells grown to the exponential phase by determining difference spectra with a Thermo Scientific BioMate 6 split beam UV/visible spectrophotometer using 1 cm light path quartz cuvettes. Particle-free solutions containing solubilized membranes and cytoplasm were obtained by adding 0.3% (w/v) of the non-ionic detergent *N,N*-dimethyldodecylamine-*N*-oxide (LDAO) to concentrated cell suspensions as outlined by Spring et al. (2009). The cytochrome *c* oxidase activity of whole cells blotted on a piece of filter paper was determined by the oxidation of *N,N,N′,N′*-tetramethyl-*p*-phenylenediamine dihydrochloride (TMPD) which results in the formation of a blue stain. Cellular fatty acid patterns were determined from cells grown to stationary phase. The preparation and extraction of fatty acid methyl esters from biomass and their subsequent separation and identification by gas chromatography was done as reported elsewhere (Miller, 1982; Kaksonen et al., 2006). Extraction and analyses of respiratory lipoquinones and polar lipids were carried out according to previously published protocols (Tindall, 1990; Tindall et al., 2007).

### Genome Sequencing and Comparative Genomics

Genomic DNA was isolated from a culture of strain L21-Syr-AB^T^ using the MasterPure Gram Positive DNA Purification Kit (Biozym Scientific, Germany) according to the manufacturer’s instructions but modified by an incubation step on ice overnight on a shaker and the use of additional 1 μl proteinase K. The culture of *D. africanus* subsp. *africanus* DSM 2603^T^ was grown anaerobically at 30°C in DSMZ medium 641^7^. Genomic DNA was isolated from this strain using the Jetflex Genomic DNA Purification Kit (Thermo Fisher Scientific, Germany) following the standard protocol provided by the manufacturer but modified by an incubation time of 60 min, the incubation on ice overnight on a shaker and the use of additional 50 μl proteinase K.

Draft genomes of the strains L21-Syr-AB^T^ and *D. africanus* subsp. *africanus* DSM 2603^T^ were generated at the DOE JGI under the umbrella of the Genomic Encyclopedia of Bacteria and Archaea (GEBA) subproject “One Thousand Microbial Genomes, phase I (KMG-I)” (Kyrpides et al., 2013, 2014). Briefly, Illumina standard shotgun libraries were constructed and sequenced with the Illumina HiSeq 2000 platform. The resulting Illumina reads were then assembled using Velvet and Allpaths-LG software. All general aspects of library construction, sequencing and assembly can be found in publications of the KMG-I consortium (e.g., Kyrpides et al., 2013; Mukherjee et al., 2015). The final assembly of the L21-Syr-AB^T^ draft genome resulted in 5 contigs, while the draft genome of DSM 2603^T^ contained 44 contigs in 41 scaffolds. Sources and assembly statistics of genomes representing strain L21-Syr-AB^T^ and closely related sulfate-reducing bacteria are shown in Supplementary Table S2. Genes were identified using Prodigal (Hyatt et al., 2010) as part of the JGI genome annotation pipeline (Huntemann et al., 2015). Genomes of additional reference strains were obtained from GenBank or IMG/M (Supplementary Table S1). Additional gene functional annotation and comparative analysis were performed within the IMG/M platform. The distribution of genes with distinct functions was analyzed using specific databases. The database TransportDB 2.0 (Elbourne et al., 2017) was used to detect membrane transporter genes, MEROPS (Rawlings et al., 2016) to determine the abundance of peptidase genes and dbCan2 (Zhang et al., 2018) to explore the distribution of genes for carbohydrate-active enzymes (Lombard et al., 2014).

Average nucleotide identity (ANI) and average amino acid identity (AAI) values among genomes were determined with the OrthoANI calculator (Lee et al., 2016) and the AAI calculator (Rodriguez-R and Konstantinidis, 2016), respectively. Synteny plots were generated by using the IMG Dotplot Synteny Viewer that employs Mummer to generate diagrams between sets of two genomes. Venn diagrams showing the shared gene content among genomes were drawn based on calculations made with the analysis option “Phylogenetic Profiler for Single Genes” available from the IMG/M system.

## Results and Discussion

### Phylogenetic Placement Within the *Desulfovibrionaceae*

**FIGURE 1:**
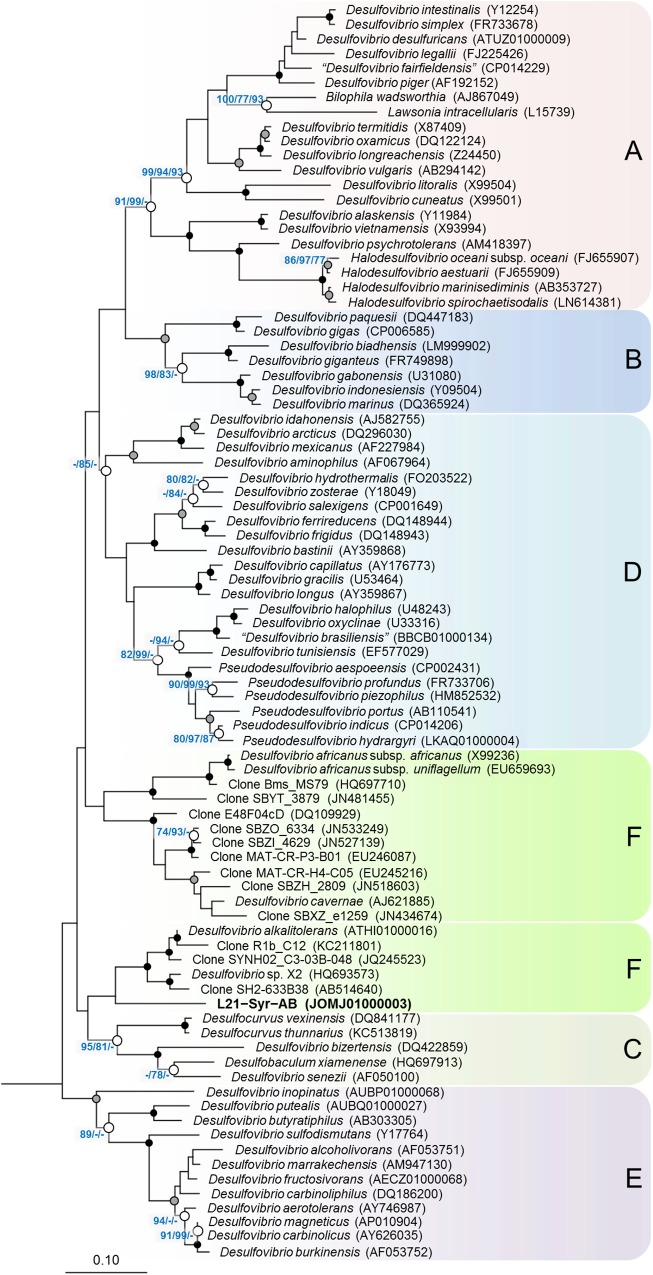
Phylogenetic tree inferred from 16S rRNA gene sequences showing the placement of strain L21-Syr-AB^T^ within the *Desulfovibrionaceae*. The tree topology was reconstructed under the maximum-likelihood criterion and rooted using the 16S rRNA gene sequences of *Desulfonatronum lacustre* (AF418171), *Desulfonatronospira thiodismutans* (EU296537), *Desulfonatronovibrio hydrogenovorans* (X99234), *Desulfovermiculus halophilus* (DQ139408), *Desulfohalobium retbaense* (CP001734), *Desulfothermus naphthae* (X80922), *Desulfonauticus submarinus* (FNIN01000014), *Desulfoplanes formicivorans* (LC017841), *Desulfomicrobium baculatum* (CP001629), and *Desulfobacter postgatei* (AGJR02000005) as outgroup (not shown). Accession numbers are given in parentheses. Support of a distinct branching by bootstrap analyses is indicated by symbols. Black dots at a distinct node indicate that bootstrap values of 95% or above (percentages of 1000 resamplings) were obtained with three different reconstruction methods, while gray dots indicate that values of 95% or above were obtained with two reconstruction methods. White dots indicate that bootstrap values of 75% or above were obtained with at least one reconstruction method. In such cases the values of 75% or above are given from left to right for the maximum-likelihood, neighbor-joining and maximum parsimony method. Letters and boxes shaded in different colors indicate distinct clades that were obtained also in trees based on the RpoB protein (Figure 2). Scale bar, 0.10 changes per nucleotide position.

Based on the comparative analysis of 16S rRNA gene sequences, strain L21-Syr-AB^T^ could be affiliated with the family *Desulfovibrionaceae* of the class *Deltaproteobacteria*, which is comprised almost exclusively of sulfate-reducing bacteria (Figure 1). There was only a distant relationship to other cultured strains within this group and the highest 16S rRNA gene identity values were shared with *Desulfovibrio alkalitolerans* DSM 16529^T^ (91.7%), *Desulfovibrio* sp. X2 (91.3%), “*Desulfovibrio cavernae”* H1M (91.2%), and *Desulfocurvus vexinense* DSM 17965^T^ (90.0%). The 16S rRNA gene identity value to *Desulfovibrio desulfuricans* DSM 642^T^, representing the type species of the genus *Desulfovibrio*, was only 88.6% and therefore significantly below the proposed cut-off level for the delineation of genera, which is usually given as 94.5% (Yarza et al., 2014). Surprisingly, there were also no closely related uncultured strains detected in the available sequence databases. Only a few environmental 16S rRNA gene sequences retrieved by cloning clustered together with strain L21-Syr-AB^T^. The most similar sequence was retrieved from a hypersaline microbial mat (JN480034) and had an identity value of only 91.6%. Further uncultured bacteria with a recognizable relationship had 16S rRNA gene identity values around 91% and were obtained from a terrestrial mud volcano (JQ245523), petroleum crude oil (AB514640) and a hot spring microbial mat (KC211801). Consequently, it appears that sulfate-reducing bacteria represented by strain L21-Syr-AB^T^ have a restricted environmental distribution and are specifically adapted to some distinct ecological niches like the suboxic zone of lithifying hypersaline cyanobacterial mats. Alternatively, it is possible that these bacteria have natural reservoirs that have rarely been screened by cultivation-independent 16S rRNA gene surveys, for instance, marine deep subsurface sites, which are considered to be under sampled (Edwards et al., 2012). An influx of groundwater from subsurface springs into Lake 21 was reported by Schneider et al. (2013) and could explain how bacteria from the subsurface could eventually get into the mat.

**FIGURE 2:**
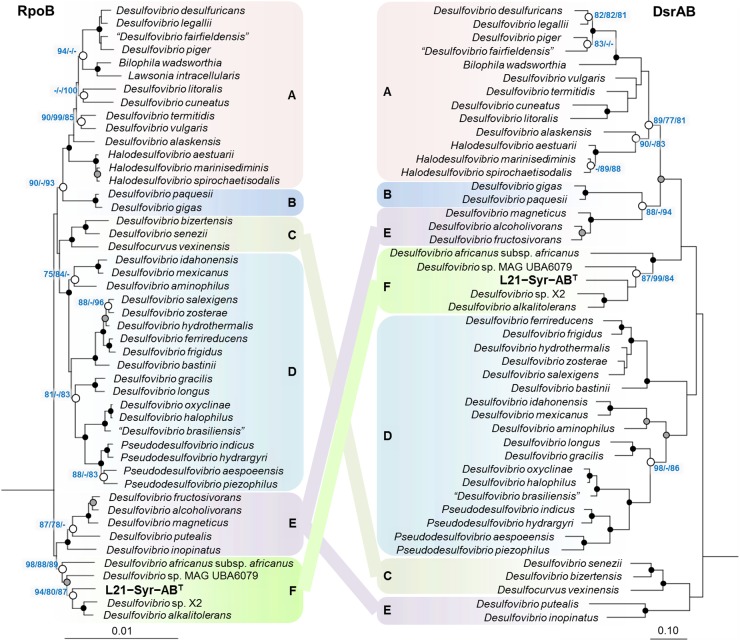
Placement of strain L21-Syr-AB^T^ within the family *Desulfovibrionaceae* based on phylogenies of complete RpoB and concatenated DsrAB protein sequences. Tree topologies were reconstructed under the maximum-likelihood criterion and rooted using the respective sequences of *Desulfonatronum lacustre*, *Desulfonatronospira thiodismutans*, *Desulfonatronovibrio hydrogenovorans*, *Desulfovermiculus halophilus*, *Desulfohalobium retbaense*, *Desulfothermus naphthae*, *Desulfonauticus submarinus*, *Desulfoplanes formicivorans*, *Desulfomicrobium baculatum*, and *Desulfobacter postgatei* as outgroup (not shown). Strains and accession numbers of genomes used for the extraction of protein sequences are provided in Supplementary Table S1. Note, that in *Bilophila wadsworthia* the *dsrB* and *dsrD* genes are combined, but for tree reconstruction the sequence of the DsrD protein was removed. Support of a distinct branching by bootstrap analyses is indicated by symbols (see caption of Figure 1). Boxes shaded in different colors indicate distinct clades supported by significant bootstrap values in the RpoB tree. Scale bars indicate changes per amino acid position.

In addition to 16S rRNA genes, protein sequences of housekeeping genes were used for the reconstruction of evolutionary relationships within the *Desulfovibrionaceae*. Large conserved proteins have in general a higher number of variable positions than 16S rRNA gene sequences, so that more reliable tree topologies are obtained. In this study, we used the beta subunit of the DNA-directed RNA polymerase (RpoB) and the concatenated alpha and beta subunits of the dissimilatory sulfite reductase (DsrAB) as alternative phylogenetic markers for tree reconstruction. The universal RpoB protein is recognized as a good proxy of the part of the genome which encodes the housekeeping genes and has been used in numerous studies to define phylogenetic relationships within various bacterial groups (e.g., Adékambi et al., 2009; Bondoso et al., 2013; Spring et al., 2015b). On the other hand, the reductive type of the Dsr protein plays an essential role in the sulfate reduction pathway and has been extensively used in studies on the environmental distribution and phylogeny of sulfate-reducing prokaryotes (e.g., Wagner et al., 1998; Müller et al., 2015; Anantharaman et al., 2018). A comparison of trees based on RpoB and concatenated reductive-type DsrAB proteins is shown in Figure 2. Tree topologies based on RpoB proteins were more reliable according to bootstrap analyses than concatenated DsrAB or 16S rRNA gene trees, which led us to use RpoB trees to define distinct clades of strains within the family *Desulfovibrionaceae*. Both RpoB and DsrAB trees show high congruence, with the exception of some inconsistencies in the branching pattern of certain clades. For instance, a group of strains which formed a common branch in trees based on RpoB proteins and 16S rRNA genes (clade E) was divided into two separate lineages in DsrAB trees. A similar result was previously obtained with a more comprehensive set of DsrAB sequences (Müller et al., 2015), which indicates that the splitting of this clade is independent of the algorithms or data sets used for tree reconstruction. In addition, some minor topological inconsistencies became apparent in the placement of clades C and F (Figure 2). The novel isolate L21-Syr-AB^T^ is included in clade F and clusters together with *Desulfovibrio alkalitolerans* and *Desulfovibrio africanus* subsp. *africanus* in RpoB and DsrAB trees, while in 16S rRNA gene trees *D. africanus* is placed along with *D. cavernae* in a separate group (Figure 1). In a recently published genome-scale tree (Parks et al., 2018)^8^ a common branch comprising the type strains of *D. africanus* subsp. *africanus* and *D. alkalitolerans* as well as strain L21-Syr-AB^T^ is supported by high bootstrap values, which is in strong agreement with the results of our phylogenetic analyses based on single proteins.

### Spatial Distribution in a Lithifying Mat

**FIGURE 3:**
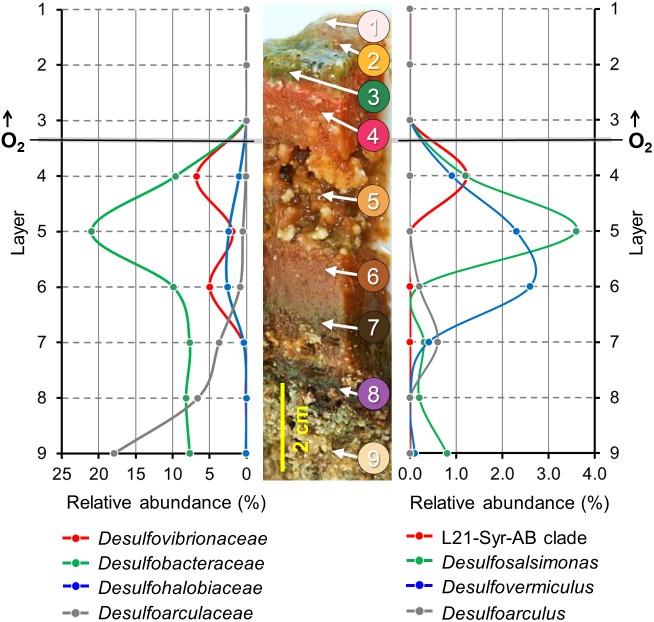
Depth profiles of prevalent groups of sulfate-reducing bacteria in a lithifying hypersaline cyanobacterial mat. Spatial distribution patterns of distinct phylogenetic clades are based on the proportion of partial 16S rRNA gene sequences in different mat layers. A representative section of the Lake 21 mat is depicted in the middle. Numbers indicate different layers of the mat used for the generation of the corresponding 16S rRNA gene sequence libraries by high-throughput pyrosequencing of amplified DNA fragments. Modified from Schneider et al. (2013). On the left, the proportion of sequences that could be affiliated with family-level clades of sulfate-reducing bacteria is plotted against different mat layers from top to bottom. On the right, the distribution patterns of the three most prevalent genera and a species-level clade comprising the novel isolate L21-Syr-AB^T^ are shown. Data points are represented by dots. Lines between the data points were extrapolated.

The Lake 21 cyanobacterial mat is characterized by different types of mineral precipitates. Large deposits of reticulate microbialites (Burne and Moore, 1987) are found at the bottom of the mat, while aragonite spherulites are mainly detected a few centimeters below the mat surface in the suboxic zone. It is assumed that the observed microbialites are formed by a slow degradation of the organic matrix in the bottom layers of the mat, while the metabolic activity of microorganisms thriving within the oxic-anoxic transition zone of the mat induces the precipitation of spherulites. Partial 16S rRNA gene sequences of high identity to the sequence of strain L21-Syr-AB^T^ were detected in a previous phylogenetic survey in the Lake 21 microbial mat near the site from which the novel isolate was obtained (Schneider et al., 2013). The results of that study were based on the pyrosequencing of 16S rRNA gene amplicons obtained from DNA extracted from discrete mat layers ranging from top to bottom, so that detailed depth profiles of certain prevalent groups of microorganisms could be obtained. The published data set was re-evaluated in the present study to localize the L21-Syr-AB^T^ clade and other known groups of sulfate-reducing bacteria in chemo-physically defined zones of the mat. The obtained depth profiles are shown in Figure 3.

Interestingly, the determined relative abundance of known sulfate-reducers is insignificant in the photic-oxic zone of the mat (Figure 3, layers 1–3) where oxygen is produced during daylight by photosynthetic cyanobacteria. This observation is in contrast to other studies reporting a peak of active sulfate-reducing bacteria in the surface layers of cyanobacterial mats (e.g., Canfield and Des Marais, 1991; Jørgensen, 1994; Teske et al., 1998). A possible reason could be that microbial mats of the Kiritimati Lake 21 had an unusual thickness of 10–15 cm leading to a greater spatial separation between the photic-oxic and permanently anoxic zone compared to most other photosynthetically-active mats studied, which typically have a stratification on the mm scale. The distribution of these bacteria in the Kiritimati mat could be more easily resolved, because on the one hand sampling of different layers was more feasible and on the other hand diurnal migration of sulfate-reducing bacteria between the surface and permanently anoxic sites of the mat are mostly negligible due to the large distances. The depth profiles of different family-level clades of sulfate-reducing bacteria within the mat were clearly distinct thereby indicating niche separation (Figure 3, left panel). Members of *Desulfobacteraceae* were the most abundant clade of sulfate-reducers and reached a maximum in layer 5, which was characterized by a disintegration of the mat matrix and large mineral aggregates (mainly gypsum). *Desulfovibrionaceae* were found mainly in layers 4 and 6, which had reddish coloration and contained calcium carbonate mineral precipitations (mainly aragonite spherulites). Sulfate-reducing bacteria affiliated with the *Desulfoarculaceae* were predominantly detected in the deep zones of the mat (layers 7–9) and were represented mostly by unclassified phylotypes. Sequences representing known genera of sulfate-reducing bacteria were most frequently associated with *Desulfosalsimonas (Desulfobacteraceae)*, *Desulfovermiculus* *(Desulfohalobiaceae)* and *Desulfoarculus (Desulfoarculaceae).* The genus *Desulfovibrio* is currently only loosely defined based on phylogenetic and phenotypic characteristics, so that its distribution and abundance was nearly identical to that of the family *Desulfovibrionaceae*. Interestingly, sequences of the L21-Syr-AB^T^ species-level clade were detected almost exclusively in layer 4 representing the suboxic transition zone of the studied mat. In most hypersaline cyanobacterial mats this zone correlates with the highest metabolic activity and represents the main site of lithification (Glunk et al., 2011; Pace et al., 2018). The proportion of sequences affiliated with the L21-Syr-AB^T^ clade was determined to be 1.23% of the total number of retrieved sequences, which corresponds to about 18% of all sequences assigned to the family *Desulfovibrionaceae*.

### Phenotypic Characterization

**FIGURE 4:**
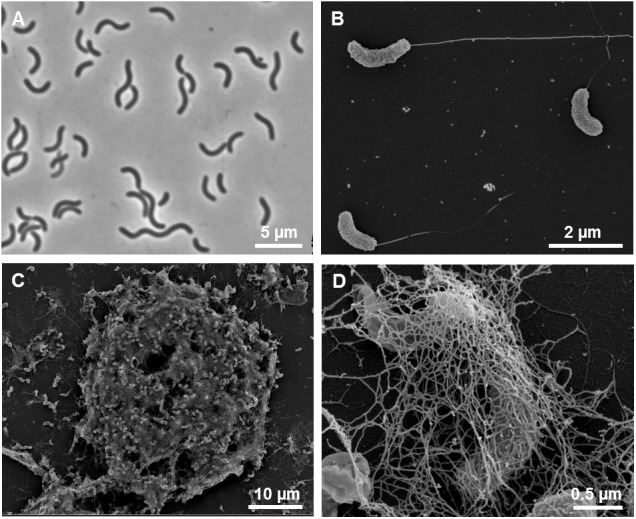
Morphological features of strain L21-Syr-AB^T^. **(A)** Phase-contrast micrograph illustrating the shape and size of cells grown in complex medium with pyruvate, sulfate and yeast extract. **(B)** Scanning electron micrograph of single cells with monopolar monotrichous flagellation. **(C)** Scanning electron micrograph of a cellular aggregate formed in defined mineral medium with pyruvate as sole source of carbon and energy. **(D)** Network of fibrillary EPS enveloping cells within aggregates.

Cells of strain L21-Syr-AB^T^ were non-spore-forming, Gram-negative, had a sigmoid or vibrio-like shape and dimensions of 0.7 μm in width and 2.5–5 μm in length. Motility was conferred by a single polar flagellum (Figure 4A,B). A morphologic peculiarity of this strain was the aggregation under conditions of nutrient limitation, which could be induced for instance by omitting yeast extract and vitamins from the medium. Nutrient deprivation first caused cells to grow attached to the glass wall of the cultivation tube thereby forming biofilms. Parts of this biofilm eventually became detached resulting in large visible aggregates that settled at the bottom of the tube. The formation of cell aggregates was obviously affected by EPS which had a net-like appearance in scanning electron micrographs and seem to connect and embed the cells (Figure 4C,D). Similar alveolar structures of EPS have previously been demonstrated in cultures of *Desulfovibrio brasiliensis* and it is assumed that nucleation sites provided by organic exopolymers may play a key role in keeping the precipitation of calcium minerals away from cells in order to prevent their entombment (Bontognali et al., 2008). A similar strategy is used by certain iron-oxidizing *Gallionella* species, which produce organic stalks to control mineral growth (Chan et al., 2011). In this case, however, the cells are not embedded in the exopolymeric matrix.

The determined optimal growth conditions of strain L21-Syr-AB^T^ reflect the habitat from which it was isolated. It is a mesophilic and moderately halophilic bacterium that grows optimally at 37°C, pH 7.5 and a salinity of 60 g/l NaCl. A broad range of salinities from 2 to 18% NaCl (w/v) is tolerated for growth, which could represent an adaptation to saline lakes exposed to alternating periods of heavy rainfall and drought. Electron donors used for growth included hydrogen, formate, ethanol, pyruvate and lactate, while acetate, propionate, butyrate, succinate, malate, fumarate, propanol, and D-fructose were not utilized. Organic substrates were incompletely oxidized to acetate. Growth on hydrogen and formate required acetate as additional carbon source. The ability to use formate and hydrogen for growth allows this strain to be part of the alkalinity engine within microbial mats which stimulates mineral precipitation by an increase of pH and calcium availability. In contrast, the consumption of organic substrates such as ethanol or lactate does not have this effect (Gallagher et al., 2012). Alternative electron acceptors besides sulfate were thiosulfate and sulfite, but elemental sulfur, selenate, arsenate, ferrihydrite, manganese (IV) oxide (MnO_2_), fumarate, nitrate, and nitrite were not utilized. Fermentative growth with pyruvate as substrate was possible and resulted in the production of acetate, CO_2_ and H_2_.

Biochemical features of strain L21-Syr-AB^T^ were determined and compared with type strains of the two most closely related species *D. alkalitolerans* and *D. africanus* subsp. *africanus*. In standard bacteriological tests all three strains were catalase positive and oxidase negative. Dithionite-reduced versus air-oxidized spectral analyses of cell-free membrane suspensions revealed a predominance of *c*-type cytochromes with γ, β, and α peaks at 419–420, 522–523, and 553 nm, respectively. The observed data would match reported absorption values of cytochrome *c*_3_, a low-molecular-weight, highly abundant soluble class III cytochrome that is characteristic for sulfate-reducing members of the *Desulfovibrionaceae* (Singleton et al., 1979). In addition, a fluorescence-emission test for the presence of desulfoviridin-type dissimilatory sulfite reductase was positive in all three strains. However, significant differences were found in regard to the compositions of respiratory lipoquinones, polar lipids and cellular fatty acids. The only detected respiratory lipoquinone in the type strain of *D. alkalitolerans* was menaquinone 7 (MK-7), while *D. africanus* subsp. *africanus* DSM 2603^T^ and strain L21-Syr-AB^T^ contained in addition MK-6 in amounts of 9.3% and 1.5%, respectively. In contrast, the predominating menaquinone in the type species of the genus *Desulfovibrio*, *D. desulfuricans*, and several closely related species is MK-6 (Collins and Widdel, 1986; Shivani et al., 2017). The major polar lipids in all three analyzed strains were phosphatidylethanolamine and phosphatidylglycerol, which is a typical feature of most species within the *Desulfovibrionaceae* (Makula and Finnerty, 1974; Shivani et al., 2017). However, strain L21-Syr-AB^T^ could be distinguished from related type strains by the presence of unidentified aminoglycolipids and an aminophospholipid, while the characteristic feature of *D. africanus* subsp. *africanus* DSM 2603^T^ was a presence of diphosphatidylglycerol (Supplementary Figure S1). Likewise, the cellular fatty acid compositions of these strains were readily distinguishable as illustrated in Supplementary Table S3. The pattern of strain L21-Syr-AB^T^ was unusual due to the complete lack of unsaturated fatty acids, which are present in most species of the *Desulfovibrionaceae* (Vainshtein et al., 1992; Shivani et al., 2017). It was further characterized by the major fatty acids *anteiso*-C_15:0_, C_16:0_ and *iso*-C_16:0_, so that it could be easily differentiated from the pattern of *D. desulfuricans* and related species which usually contain significant amounts of the fatty acid *iso*-C_17:1_ *cis*7. *D. alkalitolerans* DSM 16529^T^ was more typical for this group of sulfate-reducing bacteria and contained *iso*-C_15:0_, *anteiso*-C_15:0_ and *iso*-C_17:1_ *cis*7 as major fatty acids, while *D. africanus* subsp. *africanus* DSM 2603^T^ was quite different by containing large amounts of the unsaturated straight-chain fatty acids C_16:1_ *cis*9 and C_18:1_ *cis*11. A list of differentiating phenotypic features of the three strains compared in this study and the type strain of the type species *D. desulfuricans* is shown in Table 1.

**Table 1:** Phenotypic traits distinguishing strain L21-Syr-AB^T^ from type strains of closely related species and *Desulfovibrio desulfuricans*.

Characteristic^a^	1^b^	2	3	4
Isolation source	Hypersaline mat	Biofilm growing in alkaline waters of a district heating system	Well water	Sandy clay soil
Morphology	Sigmoid, aggregates	Vibrio	Sigmoid, spherical	Sigmoid
Cell size [μm]	0.7 × 2.5–5	0.5–0.8 × 1.4-1.9	0.5 × 5–10	0.5–0.8 × 1.5–4
Flagellation	Single polar	Single polar	Multiple polar	Single polar
Temperatures [°C]				
Range	25–42	16–47	20–40	ND
Optimum	37	43	37	30–36
NaCl conc. [g/l]				
Range	40–180	0.9–7	0–40	0–24
Optimum	40–60	1.3	0–1	0–1
pH				
Range	6.5–8.5	6.9–9.9	6.6–7.7	4.5–8.7
Optimum	7.5	9.0–9.4	7.0	7.2–7.8
Catalase	+	+ ^c^	w ^c^	– ^c^
Cytochrome *c* Oxidase	–	–^c^	– ^c^	– ^c^
Growth factor requirement	–	Yeast extract	–	–
Electron donors (with sulfate)				
Ethanol	+	–	+	+
*N*-propanol	–	–	ND	+
Malate	–	–	–	+
Fumarate	–	–	ND	+
Electron acceptors (with lactate)				
Nitrate	–	–	–	+
Sulfur	–	–	ND	–
Respiratory quinones	MK-7 (MK-6)	MK-7 ^c^	MK-7 (MK-6) ^d^	MK-6 (MK-5)
Major cellular fatty acids (>15% of total)	*ai*-C_15:0_, C_16:0_	*i*-C_15:0_, *ai*-C_15:0_^c^	C_16:1_ *c*9, C_18:1_ *c*11 ^c^	*i*-C_17:1_ *c*7, *i*-C_15:0_
Polar lipids	PE, PG, AL, GLN, PN	PE, PG, AL ^c^	PE, PG, DPG, PL, AL ^c^	ND
DNA G+C content [mol%]	65.5	64.5 ^e^	61.1 ^e^	57.4 ^e^


*^a^All strains are Gram-negative; contain desulfoviridin and cytochrome *c*_*3*_; require an organic carbon source for growth; utilize hydrogen, formate, pyruvate and lactate as electron donors and sulfate, sulfite and thiosulfate as electron acceptors; ferment pyruvate; do not utilize acetate, propionate, butyrate and fructose as substrates. ND, not determined; +, positive, -, negative; w, weakly positive; *i* and *ai* indicate *iso*- and *anteiso*-branched fatty acids, respectively; PE, phosphatidylethanolamine; PG, phosphatidylglycerol; AL, unidentified aminolipids; DPG, diphosphatidylglycerol; GLN, unidentified aminoglycolipids, PN, unidentified aminophospholipid; PL, unidentified phospholipids. ^*b*^Strains and used references: 1, L21-Syr-AB^*T*^ (this study); 2, *D. alkalitolerans* DSM 16529^*T*^ (this study, Abildgaard et al., 2006); 3, *D. africanus* subsp. *africanus* DSM 2603^*T*^ (this study, Castañeda-Carrión et al., 2010); 4, *D. desulfuricans* DSM 642^*T*^ (Vainshtein et al., 1992; Fröhlich et al., 1999; Kuever, 2014). ^*c*^Results obtained in this study. ^*d*^The respiratory quinones MK6(H2) and MK5(H2) were reported previously for strain *Desulfovibrio africanus* subsp. *africanus* NCIMB 8401^*T*^ (Collins and Widdel, 1986), which is in contrast to our results. Therefore, it is unclear if the authentic type strain was analyzed. ^*e*^Deduced from genome sequence.*

### Insights From the Genome Sequence

#### General Characteristics of the Genome

The draft genome sequence of strain L21-Syr-AB^T^ consists of five DNA scaffolds. It has a size of 3.39 Mb and a G+C content of 65.47 mol%. In total 3251 genes were predicted including 3192 protein-coding genes of which 78.6% were assigned a putative function by the IMG/M genome annotation pipeline. In addition, 59 RNA genes were detected including two complete operons for ribosomal RNA. Among protein-coding genes assigned to clusters of orthologous groups of proteins (COGs) relatively high proportions were assigned to the functional categories amino acid transport and metabolism (9.58%), energy production and conversion (8.94%) and signal transduction mechanisms (8.85%). In general, the observed distribution of genes into functional categories reflects the high capacity of sulfate-reducing bacteria to respond to environmental stimuli and their specialization on a few small organic molecules as electron donors for energy metabolism.

As expected, further analyses of genes involved in the metabolism of carbohydrates and proteins showed no indications for enzymes with a function in the degradation of extracellular macromolecules for substrate utilization. The detailed classification of carbohydrate-active proteins revealed that most of the detected genes are involved in the synthesis of intracellular carbohydrates (glycogen, trehalose), cell wall glycans or exopolysaccharides (Supplementary Table S4). A distinct region spanning 39 kb of the genome mainly contains genes involved in the synthesis of exopolysaccharides (N911DRAFT_2579 - 2610). Some genes of this cluster encode enzymes with a PEP-CTERM protein-sorting signal, which are exported by the PEP-CTERM/Exsortase system and associated with exopolysaccharide and biofilm formation in many Gram-negative bacteria (Haft et al., 2006). A polysaccharide deacetylase with a PEP-CTERM motif (N911DRAFT_2586) might be of particular interest provided it participates in the degradation of functional groups of exopolysaccharides capable of binding calcium.

Analysis of the genome sequence using the TransportDB 2.0 database revealed a large number of genes involved in membrane transport (11.2%). About half of these genes (173) represent transporters of the ATP-binding cassette (ABC) superfamily. Most of the encoded transporters have a predicted function in the uptake of osmolytes (glycine betaine, proline), essential nutrients (ammonium, phosphate, iron, lactate, sulfate) or important trace elements (tungsten, zinc). A comparison of the genetic inventory of strain L21-Syr-AB^T^ to related *Desulfovibrio* strains revealed no significant differences in the distribution of genes assigned to functional categories (Table 2). However, some differences were found in the number of transposases and non-functional genes (pseudogenes). The genome of strain L21-Syr-AB^T^ contains 70 putative transposases (2.2% of all protein-coding genes), while *D. alkalitolerans* DSM 16529^T^, strain X2 and *D. africanus* subsp. *africanus* DSM 2630^T^ encode only 5, 4 and 19 transposases, respectively. According to the IMG/M system the genome of strain L21-Syr-AB^T^ contains 68 pseudogenes, compared to only 16 in *D. alkalitolerans* and none in strain X2 and *D. africanus* subsp. *africanus*. The expansion of transposase genes and pseudogenes in genomes of strains isolated from the Kiritimati microbial mat was also noted in previous studies (Spring et al., 2016, 2018; Ben Hania et al., 2017) and thus might be a general phenomenon related to this environment. It is likely that this habitat provides isolated niches that stimulate high rates of evolution and relaxed genome structures, allowing for rapid adaptation to environmental stress while lacking stringent selective pressure to maintain genome integrity. The genomes of L21-Syr-AB^T^ and related *Desulfovibrio* strains contain one CRISPR locus each and several putative prophage regions that could play a role in genome rearrangements.

**Table 2:** Gene content analysis of genomes of strain L21-Syr-AB^T^ and related strains.

Designation	Number of genes^a^					
	Total	Transporters	CAZy	Peptidases	Transposases	Pseudo
L21-Syr-AB^T^	3251	359 (11.2)	58 (1.8)	62 (1.9)	70 (2.2)	68
*D. alkalitolerans* DSM 16529^T^	2916	303 (10.6)	46 (1.6)	53 (1.9) ^b^	5 (0.2)	16
X2	3497	379 (11.0)	61 (1.8)	55 (1.6)^b^	4 (0.1)	0
*D. africanus* subsp. *africanus* DSM 2603^T^	4062	392 (9.8)	84 (2.1)	109 (2.7)^b^	19 (0.5)	0


*^a^Numbers in brackets indicate the percentage of the total number of protein coding genes. ^*b*^Data taken from the MEROPS database.*

#### Energy Metabolism

Based on the annotated genome sequence main pathways of energy conservation in strain L21-Syr-AB^T^ could be reconstructed (Supplementary Table S5). The phenotypic characterization indicates that this strain can only use hydrogen, formate, ethanol and some organic acids as electron donors for the reduction of sulfate. In photosynthetic microbial mats, hydrogen represents a particularly important substrate for sulfate-reducers, because it is continuously produced by fermentative bacteria in anoxic zones and by nitrogen-fixing cyanobacteria in the upper layers of the mat. Three operons encoding hydrogenases were identified in the genome of strain L21-Syr-AB^T^. In comparison, *Desulfovibrio gigas* has only two hydrogenases while *Desulfovibrio vulgaris* that is assumed to use a hydrogen cycle for the generation of additional energy during growth on organic substrates encodes seven (Morais-Silva et al., 2013). The detected periplasmic [NiFe] hydrogenase HynAB is bidirectional and required for the utilization of hydrogen as substrate as well as for the production of hydrogen during fermentation of pyruvate. An energy-conserving membrane-bound [NiFe] hydrogenase (Ech complex) facing the cytoplasm could play a role in the cycling of hydrogen, while the function of a soluble hydrogenase complex (MvhADG-HdrABC) located in the cytoplasm is unclear. In methanogenic archaea this enzyme oxidizes hydrogen using electron bifurcation so that the endergonic reduction of ferredoxin with hydrogen is coupled to the exergonic reduction of the CoM-S-S-CoB heterodisulfide of methanogens (Rabus et al., 2015). The MvhADG-HdrABC complex was restricted to strain L21-Syr-AB^T^ among members of clade F shown in Figure 2. Formate as a substrate is cleaved into carbon dioxide and protons by a soluble enzyme within the periplasm. Two sets of genes were detected encoding formate dehydrogenases (FdhAB) comprising a molybdenum cofactor- and an iron-sulfur cluster-containing subunit. Both subunits are likely targeted to the periplasm as a complex by the twin-arginine transport (Tat) pathway. The electrons released in the periplasm by formate dehydrogenase and HynAB hydrogenase are transferred to a type I cytochrome *c*_3_ (CycA).

**FIGURE 5:**
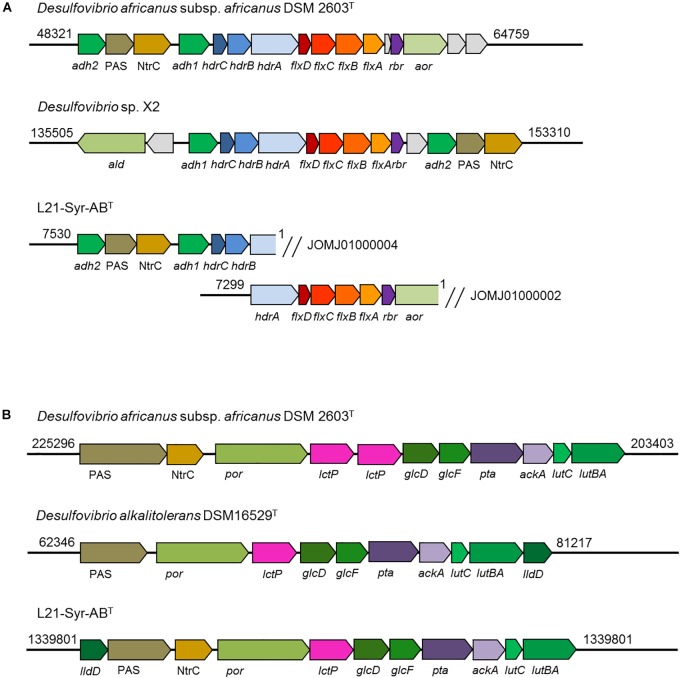
Organization of genomic regions involved in ethanol and lactate utilization in strain L21-Syr-AB^T^ and related strains. The genomic organization was assessed using the IMG/M system. Arrows show gene direction and relative size. Similar functions of genes are indicated by related colors. Explanation of gene symbols can be found in Supplementary Table S5. Numbers at the beginning and end of the genomic region denote nucleotide positions of the respective genome sequence. **(A)** Organization of genes involved in ethanol utilization. Note that the ethanol utilization genes of strain L21-Syr-AB^T^ were distributed in two different contigs. **(B)** Organization of genes involved in lactate and pyruvate utilization.

A NADH dehydrogenase/heterodisulfide reductase complex (FlxABCD-HdrABC) is likely involved in the utilization of ethanol in strain L21-Syr-AB^T^ as was shown previously in *Desulfovibrio vulgaris* strain Hildenborough (Ramos et al., 2015). In *D. vulgaris* an alcohol dehydrogenase gene (*adh1*) is linked to the *flxABCD-hdrABC* genes in a single operon. It is proposed that NADH produced by the alcohol dehydrogenase is oxidized by the Flx dehydrogenase that transfers electrons to the heterodisulfide reductase which in turn reduces ferredoxin (endergonic) and DsrC (exergonic) using an electron bifurcation mechanism. In a study by Ramos et al. (2015) it is proposed that the Flx-Hdr complex may also operate in reverse and participate in the reduction of acetyl-CoA to ethanol during pyruvate fermentation, for example, when the more energy efficient production of hydrogen is inhibited. Similar gene arrangements were observed in strain L21-Syr-AB^T^, *D. africanus* subsp. *africanus* DSM 2603^T^ and strain X2, but not in *D. alkalitolerans* DSM 16529^T^ which is not able to utilize ethanol (Figure 5A). In the genomes of L21-Syr-AB^T^ and related strains capable of using ethanol, genes of this complex are associated with an aldehyde ferredoxin oxidoreductase gene (*aor*) and a gene encoding a second alcohol dehydrogenase (*adh2*). The *adh2* gene is followed by two genes, one encoding a PAS domain-containing sensor histidine kinase and the other is an NtrC family DNA-binding transcriptional response regulator.

Genes involved in the utilization of pyruvate and lactate were organized in a cluster (Figure 5B). This genomic region contains genes of a pyruvate:ferredoxin oxidoreductase (*por*), lactate permease (*lctP*), two subunits of a putative D-lactate dehydrogenase (*glcFD*), phosphate acetyltransferase (*pta*) and acetate kinase (*ackA*). Adjacent to this gene cluster a potential lactate dehydrogenase has been detected that is encoded by the *lutABC* genes, which have been linked to the utilization of lactate in a wide range of diverse bacteria (Hwang et al., 2013). In strain L21-Syr-AB^T^ and related sulfate-reducing bacteria *lutB* and *lutA* are combined in a single gene. A further putative FMN-dependent L-lactate dehydrogenase is located close to a PAS domain-containing sensor histidine kinase and an NtrC family DNA-binding transcriptional response regulator that probably control the expression of this gene cluster in a similar way as the genes involved in ethanol utilization. The possible presence of a diverse set of lactate dehydrogenases in this clade of sulfate-reducing bacteria may be explained by different substrate affinities of the respective enzymes or the use of various electron acceptors. It is assumed that in contrast to the oxidation of ethanol, pyridine nucleotides are not involved in the oxidation of lactate to pyruvate (Rabus et al., 2015), so that ferredoxin and DsrC or cytochromes might be reduced directly by one of the lactate dehydrogenases using an electron bifurcation mechanism.

The preferred terminal electron acceptor for the oxidation of substrates in strain L21-Syr-AB^T^ is sulfate which is reduced to sulfide. Genes encoding various potential sulfate transporters are present in the genome including three distinct sulfate permeases of which one (*sulP2*, N911DRAFT_1570) is located close to a cluster of genes involved in sulfite reduction (N911DRAFT_1528-1566). However, there is limited knowledge about the type of transporters used in sulfate-reducers for the uptake of sulfate (Marietou et al., 2018). Genes required for the reduction of sulfate to sulfite are located in a separate cluster at N911DRAFT_0238-0244. The gene of the sulfur relay protein DsrC (N911DRAFT_0568) which transfers electrons from the DsrMKJOP membrane complex to the dissimilatory sulfite reductase DsrAB is not linked to any other gene involved in sulfate reduction.

Besides substrate-level phosphorylation (mainly based on acetate kinase, N911DRAFT_1390), most sulfate-reducing bacteria should be able to gain energy by a chemiosmotic potential generated by several electron-transfer membrane complexes (Rabus et al., 2015). The putative ion-translocating membrane-bound complexes QmoABC and DsrMKJOP are directly involved in the sulfate-reduction pathway and transfer electrons to soluble adenylsulfate reductase and dissimilatory sulfite reductase, respectively, probably using menaquinol (MQH2) as reductant. In addition, the Ech hydrogenase and the ferredoxin:NAD^+^ oxidoreductase (Rnf complex) could participate in the generation of a chemiosmotic potential by using ferredoxin as electron donor for the oxidation of protons or NAD^+^, respectively. The electron donor used by a Nuo (NADH:ubiquinone oxidoreductase)-like complex for menaquinone reduction and proton translocation is unknown, but it is likely also ferredoxin (Rabus et al., 2015). The function of further electron-transfer complexes is more obscure and their presence may vary among different species of sulfate-reducing bacteria (Table 3). Interestingly, the genome of *D. alkalitolerans* lacks genes of an Rnf complex while it encodes Nhc and Hmc complexes that are missing in other representatives of this clade. The Ohc complex was only detected in strain L21-Syr-AB^T^. Presumably, the chemiosmotic potential generated during sulfate-reduction is used for the production of ATP by an F-type ATP synthase complex, while the V-type ATPase could be involved in cytoplasmic pH homeostasis.

**Table 3:** Membrane-bound electron-transfer complexes in strain L21-Syr-AB^T^ and related strains.

ETF complex	Assumed redox partners	L21-Syr-AB^T^	*D. alkalitolerans* DSM 16529^T^	X2	*D. africanus* subsp. *africanus* DSM 2603^T^
EchABCDEF	Fd/H^+^(H_2_)	+	+	+	+
NuoABCDHIJKLMN	Fd?/MQ	+	+	+	+
RnfABCDEG	Fd/NAD(H)	+	–	+	+
QmoABC	MQH_2_/AprAB	+	+	+	+
DsrMKJOP	MQH_2_/DsrC	+	+	+	+
QrcABCD	TpIc_3_/MQ	+	+	+	+
OhcABB2C	TpIc_3_/MQ	+	-	-	-
NhcABCD	TpIc_3_/MQ	-	+	-	-
TmcABCD	TpIc_3_/DsrC	+	+	+	+
HmcABCDE	TpIc_3_/DsrC	-	+	-	-


#### Protection Against Osmotic and Oxidative Stress

The salinity of hypersaline lakes on the Kiritimati Atoll can vary widely due to cyclic climatic phenomena such as El Niño, which cause alternating periods of heavy rains and drought. Microorganisms that inhabit photosynthetically active mats of these lakes are therefore frequently exposed to environmental stress factors such as hyper- or hypoosmotic shock, desiccation or oxidative damage caused by the oxygen production of cyanobacteria in the upper layers of the mat. The main protection mechanism against salt stress and desiccation in bacteria is the accumulation of compatible solutes. Based on the gene content analysis of the L21-Syr-AB^T^ genome the osmolytes proline, glycine betaine, and trehalose could play a role in this strain. Glycine betaine or its precursor choline is taken up from the environment via specific transport proteins, proline can be imported or synthesized and the disaccharide trehalose is mainly produced within the cytoplasm. External glycine betaine is probably imported by specific transporters of the ABC family while transporters of the BCCT family (*opuD*, N911DRAFT_0045 and N911DRAFT_2495) could also transport choline using an ion-motive force. Choline can be further oxidized to glycine betaine in the cytoplasm by the enzymes choline dehydrogenase (N911DRAFT_2554) and betaine-aldehyde dehydrogenase (N911DRAFT_2553). Transporter of the sodium:solute symporter (SSS) family are probably responsible for the import of proline and a search against the TransportDB 2.0 database revealed twelve genes possibly encoding sodium ion:proline symporter (e.g., N911DRAFT_1471), which points to a possible role of this compound as osmolyte. Alternatively, strain L21-Syr-AB^T^ should be able to synthesize proline using pathways of its amino acid metabolism. Several pathways are likely involved in the intracellular metabolism of trehalose. In the presence of the storage polysaccharide glycogen trehalose can be synthesized from maltodextrins by the enzymes malto-oligosyltrehalose synthase (*treY*, N911DRAFT_3179) and malto-oligosyltrehalose trehalohydrolase (*treZ*, N911DRAFT_3181) while the enzyme trehalose synthase *(treS*, N911DRAFT_1768) converts maltose to trehalose (Ruhal et al., 2013). A third pathway proceeds from glucose-6-phosphate and UDP-glucose to yield trehalose 6-phosphate which is then dephosphorylated to trehalose. The involved enzymes are trehalose 6-phosphate synthase (*otsA*, N911DRAFT_0985) and trehalose-6-phosphate phosphatase (*otsB*). Interestingly, in this clade of sulfate-reducing bacteria the *otsB* gene is difficult to detect. In *D. africanus* subsp. *africanus* DSM 2603^T^ a phosphatase region is fused with a trehalose hydrolase domain (*treH*) resulting in a large protein of more than 1095 amino acids (*otsAB*, H585DRAFT_00220), while in a similar protein of strain L21-Syr-AB^T^ (N911DRAFT_1271) only the hydrolase, but not the phosphatase was recognized. It is possible that this enzyme has a regulatory function and participates in the immediate control of the cytoplasmic trehalose concentration.

Sulfate-reducing bacteria have developed several strategies to avoid cellular damage caused by the contact with oxygen. The genome of strain L21-Syr-AB^T^ encodes a membrane-bound cytochrome *bd* ubiquinol oxidase (*cydAB*, N911DRAFT_2321 and 2322) that reduces oxygen to water, but no haem-copper cytochrome *c* oxidase (Cox), which has been detected in several *Desulfovibrio* species (Rabus et al., 2015). Within the cytoplasm oxygen can be reduced by a rubredoxin:oxygen oxidoreductase (*roo*, N911DRAFT_2325) that receives electrons from NADH via rubredoxin (*rub*, N911DRAFT_2326). However, the actual oxidative stress is caused by reactive oxygen species (ROS), which are formed by contact with cellular redox enzymes. Superoxide can be inactivated either by a cytoplasmic desulfoferrodoxin-type superoxide reductase (*dfx*, N911DRAFT_2327) that reduces superoxide to H_2_O_2_ with rubredoxin as electron donor or by a periplasmic superoxide dismutase (*sod*, N911DRAFT_2660) that produces oxygen and H_2_O_2_. In addition, neelaredoxin (*nlr*, N911DRAFT_3205) might function as cytoplasmic superoxide dismutase. The produced hydrogen peroxide is then destroyed by several enzymes including a membrane-bound cytochrome *c* peroxidase (*ccp*, N911DRAFT_2926) or the cytoplasmic proteins catalase (*katA*, N911DRAFT_1613) and rubrerythrin (*rbr*, N911DRAFT_1786).

Certain toxic trace minerals, especially arsenic and mercury, can occur in significant quantities in natural environments and may cause severe damage to living cells due to the generation of ROS or binding to the active sites of enzymes. The source of arsenic in hypersaline lakes on Kiritimati is most likely the inflow of groundwater from underground springs (Schneider et al., 2013). In cells of strain L21-Syr-AB^T^ arsenite, As(III), can be exported by an arsenite efflux pump (*arsB*, N911DRAFT_2477), while the less toxic arsenate, As(V), has to be first reduced to arsenite by an arsenate reductase (*arsC*, N911DRAFT_2457). Genes involved in arsenic metabolism are frequently found in metagenomes derived from both freshwater and hypersaline microbial mats (Ruvindy et al., 2016; White et al., 2016). In addition, several isolates retrieved from stromatolites were shown to be extremely tolerant to arsenic (Gorriti et al., 2014). The accumulation and cycling of arsenic by heterotrophic bacteria was probably an important function in ancient stromatolites exposed to high concentrations of arsenic due to volcanic activity (Sforna et al., 2014). One could speculate that in this way the overlying waterbody was effectively protected from toxic arsenic concentrations so that less tolerant cyanobacteria were able to thrive in these environments.

The toxic heavy metal mercury enters pristine aquatic environments mainly through the atmosphere or terrestrial run-offs. Complexing thiols facilitate the accidental uptake of environmental Hg(II) via an active transport mechanism that is probably actually intended for transporting essential trace elements (Schaefer et al., 2011). In sulfate-reducing bacteria the build-up of Hg(II) in the cytoplasm is avoided by methylation to methylmercury (CH_3_Hg^+^) that is subsequently exported. Recent genetic studies linked the presence of the genes *hgcA* and *B* with the methylation of mercury in sulfate-reducing bacteria (Brown et al., 2013; Parks et al., 2013). Both genes could be also detected in strain L21-Syr-AB^T^. However, in this strain the *hgcA* gene has been split into two open reading frames (N911DRAFT_0641 and 0642), which are both located next to the *hgcB* gene (N911DRAFT_0643) encoding a 2[4Fe–4S] ferredoxin.

### Possible Stimulation of Calcification by Strain L21-Syr-AB^T^

Sulfate-reducing bacteria are very common in hypersaline microbial mats and have been implicated in the lithification process due to their physiological properties and vertical distribution. However, sulfate-reducing bacteria represent a very diverse group with a range of metabolic traits, so it is important to identify potential key players at the species level. Based on the results obtained in this study, we suggest that sulfate-reducing bacteria represented by the strain L21-Syr-AB^T^ might play a role in the precipitation of carbonates in microbial mats of the hypersaline Kiritimati Lake 21. One reason for this assumption is the restricted geographic and spatial distribution of this species, limited to the suboxic zone of a hypersaline mat characterized by the precipitation of aragonite. It should be noted, however, that the thick, gelatinous microbial mats of Kiritimati Lake 21 are rarely found in other parts of the world, which may also contribute to the endemic occurrence of this species. Sulfate-reducing bacteria similar to L21-Syr-AB^T^ have the potential to release free calcium that is complexed with small organic compounds or bound to functional groups of the mat matrix by the consumption of organic acids (mainly lactate) and the deacetylation of polysaccharides by extracellular carbohydrate esterases. On the other hand, utilization of hydrogen and formate by strain L21-Syr-AB^T^ contributes to the alkalinity engine within the mat thereby promoting precipitation of calcium carbonate. The formation of cell aggregates embedded in extracellular polymers represents a special feature of strain L21-Syr-AB^T^ that could be involved in the lithification process. The EPS formed by this strain could provide a template for aragonite formation away from the cell surface, allowing living cells to escape entombment.

However, it should be kept in mind that the processes leading to the precipitation of calcium carbonate in microbial mats are likely to be very complex and not due to a single type of metabolism. To gain a deeper understanding of the contribution of sulfate-reducing bacteria to the calcification process within this mat, future studies would be needed. Above all, it would be necessary to define the precise growth conditions that lead to the precipitation of calcium carbonate in cultures of strain L21-Syr-AB^T^. Then the optimum conditions for the carbonate precipitation could be compared with the microenvironment found in the mat. In addition, it would be possible to determine the amount and quality (mineral type) of the CaCO_3_ formed and to compare it with the carbonate minerals identified in the mat.

### Taxogenomics and Classification

Genome sequences can offer valuable insights for the classification of microorganisms in addition to the traditionally used phenotypic traits. Genome-based tools that rely on the determination of ANI and average AAI values are now widely accepted for the definition of species and genera. The threshold used for the demarcation of species is around 95% ANI/AAI, while for the delineation of genera AAI values in the range of 60–65% are recommended (Rodriguez-R and Konstantinidis, 2014; Konstantinidis et al., 2017). It turned out that the cut-off value used for the definition of species is very accurate and reliable, while it is more difficult to correlate an exact AAI value with the established genus-level taxonomy of bacteria and archaea (Qin et al., 2014). This is in part due to the fact that early in microbiology DNA-DNA hybridization assays were used to assign strains to certain species, while the definition of genera was mainly subjective and initially relied on only a few selected phenotypic traits. Only recently, it was proposed to use a threshold of 94.5% 16S rRNA gene identity for members of a single genus which should form a monophyletic group in reconstructed 16S rRNA gene trees (Yarza et al., 2014). However, this approach has also some limitations because the classification relies on a single gene with only a limited number of variable positions available for phylogenetic analyses. ANI and AAI values determined between the genomes of L21-Syr-AB^T^ and related strains as well as the type strain of *D. desulfuricans* are shown in Table 4. It is obvious that all genomes represent different species due to the obtained ANI values below 95% and the low 16S rRNA gene identity values. All determined AAI values were below 65%, but below 60% only with the type strains of *D. africanus* subsp. *africanus*, *Desulfocurvus vexinensis* and *D. desulfuricans*. Hence, for the reliable demarcation of genera within this clade of sulfate-reducers further genome analyses are required. An alternative approach is based on the determination of the shared gene content among genomes, which has the advantage that environmental factors that control the evolutionary niche adaptation and thus the genetic inventory of species are taken into consideration. A recent study suggested that the percentage of conserved proteins (POCP) among genomes of members of a single genus should be above 50% (Qin et al., 2014). In Supplementary Figure S2 Venn diagrams illustrating the overlap of protein-coding genes among strains of the L21-Syr-AB^T^ clade and *D. desulfuricans* are shown. The novel isolate L21-Syr-AB^T^, *D. alkalitolerans* DSM 16529^T^ and the unclassified strain X2 share 2100 homologous proteins and the overlap of the total protein-coding genes between two of each genomes is around 70%, which suggests an affiliation of these strains to a single genus. A comparison with *D. africanus* subsp. *africanus* DSM 2603^T^ resulted in a shared protein-gene content of 55 and 51% with the strains L21-Syr-AB^T^ and *D. alkalitolerans* DSM 16529^T^, respectively. Although, slightly above the recommended cut-off value the large number of 1604 unique proteins in *D. africanus* subsp. *africanus* DSM 2603^T^ could be an indication for placement of this species in a separate genus. As expected, the shared protein content between the type strain of *D. desulfuricans* and both strains L21-Syr-AB^T^ and *D. alkalitolerans* DSM 16529^T^ was below 50%. The increase of evolutionary divergence among these strains is also illustrated in synteny plots shown in Supplementary Figure S3. Between the strains L21-Syr-AB^T^, X2 and *D. alkalitolerans* DSM 16529^T^ continuous clusters of syntenic genes can be detected. While still small syntenic blocks could be detected between strains L21-Syr-AB^T^ and *D. africanus* subsp. *africanus* DSM 2603^T^, no synteny is recognizable with the genome of *D. desulfuricans* DSM 642^T^.

**Table 4:** Sequence identity values of genomes, total predicted proteins and phylogenetic marker genes between the novel isolate L21-Syr-AB^T^ and related strains or MAGs.

Strain or MAG used for comparison	AAI	ANI	*rpoB* gene	*dsrAB* gene	16S rRNA gene
*Desulfovibrio alkalitolerans* DSM 16529^T^	61.7^a^	72.3	81.6	86.0	91.7
X2	60.5	72.6	82.2	86.1	91.3
MAG UBA6079^b^	57.1	70.9	82.1	84.6	– ^c^
*Desulfovibrio africanus* subsp. *africanus* DSM 2603^T^	56.3	69.7	77.6	81.4	89.1
*Desulfocurvus vexinensis* DSM 17965^T^	56.0	71.5	79.7	81.3	90.0
*Desulfovibrio desulfuricans* DSM 624^T^	48.5	67.0	75.4	76.5	88.6


*^a^Values are given in percent. ^*b*^Values are based on a metagenome-assembled genome (MAG). ^*c*^Value could not be determined because the MAG does not contain a 16S rRNA gene.*

Sequences of certain housekeeping genes can be used as proxy of the conserved part of the core genome thereby enabling a demarcation of genera. The gene of the RNA polymerase beta subunit (*rpoB*) has been widely used to define taxonomic ranks in various groups of bacteria. In previous studies the threshold for the delineation of genera was empirically determined to be around 85% identity of the complete *rpoB* gene sequence (e.g., Adékambi et al., 2008; Spring et al., 2013). As shown in Table 4 identity values of the L21-Syr-AB^T^ *rpoB* gene to the sequences of related type strains were well below 85% thereby suggesting the proposal of a separate genus represented by strain L21-Syr-AB^T^. In contrast to the *rpoB* gene, the distribution of *dsrAB* genes is essentially restricted to sulfate-reducing prokaryotes. Applying a 16S rRNA gene identity of 94.5% for the delineation of genera to sequence identity plots of 16S rRNA and concatenated *dsrAB* genes (Müller et al., 2015) results in a threshold of 85% *dsrAB* gene identity. Therefore, based on the comparison of *dsrAB* sequences the strains L21-Syr-AB^T^, X2 and *D. alkalitolerans* DSM 16529^T^ could be assigned to a single genus (Table 4), which would confirm the results of the gene content analyses and the application of an AAI value of 60% for the demarcation of genera. Consequently, in this clade of sulfate-reducing bacteria, the evolution of the overall genome structure and content is more accurately represented by genes involved in central metabolic pathways than by genes of the protein synthesis machinery that might evolve faster than usual. In summary, a threshold of 85% *dsrAB* gene identity and an AAI value of 60% proved to be the best approaches for genus demarcation in this group of bacteria, while established 16S rRNA and *rpoB* gene identity values appear less suitable.

Based on the presented results, a novel species, represented by the isolate L21-Syr-AB^T^, is proposed and assigned to the novel genus *Desulfohalovibrio.* The species *D. alkalitolerans* should be reclassified and affiliated to the genus *Desulfohalovibrio*. Furthermore, the neighboring species *D. africanus* should be placed in the novel genus *Desulfocurvibacter*. A distinction of both genera is possible based on the following phenotypic traits: NaCl is a requirement for growth in *Desulfohalovibrio* strains, but not *Desulfocurvibacter*; unsaturated cellular fatty acids are predominating in *Desulfocurvibacter* strains while branched-chain fatty acids dominate in *Desulfohalovibrio* cells; the polar lipid diphosphatidylglycerol was detected so far only in *Desulfocurvibacter*. In addition, it can be noted that type strains of both *Desulfohalovibrio* species were isolated from microbial communities that formed laminated structures (Table 1). The results of our polyphasic study are also supported by the classification proposed in the Genome Taxonomy Database^9^, which is based on the relative evolutionary divergence deduced from the phylogeny of 120 conserved single-copy proteins (Parks et al., 2018). Formal descriptions of the suggested novel taxa follow below:

#### Description of *Desulfohalovibrio* gen. nov.

*Desulfohalovibrio* [De.sul.fo.ha.lo.vi’bri.o. L. pref. *de*, from; L. n. *sulfur*, sulfur; N.L. pref. *desulfo-*, desulfuricating (prefix used to characterize a dissimilatory sulfate-reducing prokaryote); Gr. n. *hals halos*, salt; L. v. *vibro*, to set in tremulous motion, move to and fro, vibrate; N.L. masc. n. *vibrio*, that which vibrates, and also a bacterial genus name of bacteria possessing a curved rod shape (*Vibrio*); N.L. masc. n. *Desulfohalovibrio*, a salt (-loving) vibrio that reduces sulfur compounds].

The description is based on this study and the characterization of *Desulfovibrio alkalitolerans* by Abildgaard et al. (2006).

Free-living, Gram-negative, non-spore-forming, unpigmented, curved to sigmoid-shaped cells occurring single or in pairs. Motility is conferred by flagella. Obligately anaerobic and mesophilic. NaCl is required for growth. Growth requires an organic carbon source. Sulfate, sulfite and thiosulfate are reduced to sulfide. Fumarate, nitrate, ferric iron or sulfur are not used as electron acceptors. Pyruvate can be fermented. Organic substrates are incompletely oxidized to acetate. Tests for catalase are positive and for cytochrome *c* oxidase negative. Cells contain desulfoviridin and cytochrome *c*_3_. Major cellular fatty acids are *anteiso*-C_15:0_, *iso*-C_15:0_, and C_16:0_. The polar lipid composition is dominated by phosphatidylethanolamine, phosphatidylglycerol and aminolipids. The main respiratory lipoquinone is menaquinone 7 (MK-7). Sequence identity values of concatenated complete *dsrAB* genes of newly described strains assigned to this genus should be around 85% or above to the corresponding sequence of the type strain of the type species.

The type species is *Desulfohalovibrio reitneri*.

#### Description of *Desulfohalovibrio reitneri* sp. nov.

*Desulfohalovibrio reitneri* [reit’ne.ri. N.L. masc. gen. n. *reitneri*, of Reitner, named to honor Prof. Dr. Joachim Reitner (University of Göttingen, Germany) for his contributions to the geobiology of hypersaline microbial mats].

Shows the following characteristics in addition to those given for the genus. Most cells have a diameter around 0.7 μm and a length ranging from 2.5 to 5.0 μm. Motile by a single polar flagellum. Nutrient deprivation causes the production of extracellular polymers and the formation of large cell aggregates. Optimal conditions for growth are 37°C, pH 7.5 and a salinity of 4 – 6% (w/v) NaCl; temperatures from 25 to 42°C and salinities of up to 180 g/l NaCl are tolerated. Vitamins or yeast extract are not required for growth. The following electron donors are utilized: hydrogen, formate, ethanol, pyruvate, and lactate. No growth in the presence of sulfate occurs with acetate, propionate, butyrate, succinate, malate, fumarate, propanol, and D-fructose. Selenate, arsenate, manganese (IV) oxide and nitrite are not reduced. Fermentation of pyruvate results in the production of acetate, CO_2_ and H_2_. Small amounts of menaquinone 6 (MK-6) can be detected in addition to the main respiratory lipoquinone MK-7. In addition to the dominating cellular fatty acids listed in the description of the genus, significant amounts (>5% of total amount) of *iso*-C_16:0_ and *anteiso*-C_17:0_ are present. The DNA G + C content of the type strain is 65.5 mol%.

The type strain, L21-Syr-AB^T^ (=DSM 26903^T^ = JCM 18662^T^), was isolated from the suboxic zone of a lithifying cyanobacterial mat in the hypersaline Lake 21 at the Kiritimati Atoll, Republic of Kiribati.

#### Description of *Desulfohalovibrio* *alkalitolerans* comb. nov.

*Desulfohalovibrio* *alkalitolerans* [al.ka.li.to’le.rans. N. L. n. *alkali* (from Arabic article *al*, the; Arabic n. *qaliy*, ashes of saltwort) alkali; L. pres. part. *tolerans* tolerating; N.L. part. adj. *alkalitolerans* alkali-tolerating].

Basonym: *Desulfovibrio alkalitolerans* (Abildgaard et al., 2006).

The description of Abildgaard et al. (2006) is amended with results obtained in this study.

In addition to traits given for the genus the following characteristics were determined. Most cells have a diameter of 0.5 – 0.8 μm and a length ranging from 1.4 to 1.9 μm. Motile by a single polar flagellum. Alkaliphilic. Optimal conditions for growth are 43°C, pH 9.0 – 9.4 and a salinity of 0.13% (w/v) NaCl; temperatures from 16 to 47°C and pH values between 6.9 and 9.9 are tolerated. Yeast extract required for growth. The following electron donors are utilized: hydrogen, formate, pyruvate, and lactate. No growth in the presence of sulfate occurs with acetate, propionate, butyrate, succinate, malate, fumarate, oxamate, citrate, benzoate, methanol, ethanol, glycerol, propanol, butanol, choline, xylose, glucose, fructose, galactose, lactose, maltose, raffinose, and sucrose. In addition to the dominating cellular fatty acids listed in the description of the genus, significant amounts (>5% of total amount) of *iso*-C_17:1_ *cis*7, *iso*-C_17:0_ and *anteiso*-C_17:1_ *cis*7 are present. The DNA G + C content of the type strain is 64.5 mol%.

The type strain, RT2^T^ (=DSM 16529^T^ = JCM 12612^T^), was isolated from mild steel coupons from a reactor connected to the return line of the Skanderborg district heating plant (Jutland, Denmark).

#### Description of *Desulfocurvibacter* gen. nov.

*Desulfocurvibacter* [De.sul.fo.cur.vi.bac’ter. L. pref. *de*, from; L. n. *sulfur*, sulfur; N.L. pref. *desulfo-*, desulfuricating (prefix used to characterize a dissimilatory sulfate-reducing prokaryote); L. adj. *curvus* curved; N.L. masc. n. *bacter* rod; N.L. masc. n. *Desulfocurvibacter* a curved sulfate-reducing rod].

Free-living, Gram-negative, non-spore-forming, unpigmented, sigmoid-shaped cells occurring single or in pairs. Spherical bodies can be formed in stationary phase cultures. Motility is conferred by flagella. Obligately anaerobic, neutrophilic and mesophilic. NaCl is not required for growth. Growth requires an organic carbon source. Sulfate, sulfite and thiosulfate are reduced to sulfide. Nitrate is not used as an electron acceptor. Pyruvate can be fermented. Organic substrates are incompletely oxidized to acetate. Tests for catalase are weakly positive and for cytochrome *c* oxidase negative. Cells contain desulfoviridin and cytochrome *c*_3_. Major cellular fatty acids are C_16:1_ *cis*9 and C_18:1_ *cis*11. The polar lipid composition is dominated by phosphatidylethanolamine, phosphatidylglycerol and diphosphatidylglycerol. The main respiratory lipoquinone is menaquinone 7 (MK-7). Sequence identity values of concatenated complete *dsrAB* genes of newly described strains assigned to this genus should be around 85% or above to the corresponding sequence of the type strain of the type species.

The type species is *Desulfocurvibacter africanus.*

#### Description of *Desulfocurvibacter africanus* omb. nov.

*Desulfocurvibacter africanus* (a.fri.ca’nus. L. masc. adj. *africanus* pertaining to Africa).

Basonym: *Desulfovibrio africanus* (Campbell et al., 1966) (Approved Lists 1980).

The description is based on the previous studies of Campbell et al. (1966) and Castañeda-Carrión et al. (2010) amended with data from this work.

In addition to traits given for the genus the following characteristics were determined. Most cells have a diameter of 0.5 μm and a length ranging from 3.5 to 10 μm. Yeast extract is not required for growth. The following electron donors are utilized: hydrogen, formate, ethanol, pyruvate, and lactate. No growth in the presence of sulfate occurs with acetate, propionate, butyrate, malate, methanol, choline, glucose, and fructose. The DNA G + C content of the type strain is 61.1 mol%.

The species is subdivided into two subspecies.

The type strain, Benghazi^T^ (=DSM 2603^T^ = ATCC 19996^T^, =NCIMB 8401^T^, =VKM B-1757^T^), was isolated from well water from Benghazi, Libya.

#### Description of *Desulfocurvibacter africanus* subsp. *africanus* comb. nov.

*Desulfocurvibacter africanus* subsp. *africanus* (a.fri.ca’nus. L. masc. adj. *africanus* pertaining to Africa).

Basonym: *Desulfovibrio africanus* subsp. *africanus* (Campbell et al., 1966; Castañeda-Carrión et al., 2010).

The description is as for *Desulfovibrio africanus* subsp. *africanus* (Castañeda-Carrión et al., 2010).

The type strain, Benghazi^T^ (=DSM 2603^T^ = ATCC 19996^T^, =NCIMB 8401^T^, =VKM B-1757^T^), was isolated from well water from Benghazi, Libya.

#### Description of *Desulfocurvibacter africanus* subsp. *uniflagellum* comb. nov.

*Desulfocurvibacter africanus* subsp. *uniflagellum* [uni.fla.gel’lum. L. adj. *unus* only one, one; L. n. *flagellum* whip; N.L. neut. n. (nominative in apposition) *uniflagellum* the only one whip].

Basonym: *Desulfovibrio africanus* subsp. *uniflagellum* (Castañeda-Carrión et al., 2010).

The description is as for *Desulfovibrio africanus* subsp. *uniflagellum* (Castañeda-Carrión et al., 2010).

The type strain, SR-1^T^ (=JCM 15510^T^ = KCTC 5649^T^ = DSM 23860^T^), was isolated from subsurface sediments of a uranium-contaminated site in Shiprock, NM, United States.

## Author Contributions

SS isolated strain L21-Syr-AB^T^, designed the study, and performed comparative genome analyses. SS and DS did the physiological characterization of pure cultures. SV supervised the determination of chemotaxonomic traits. MR performed electron microscopy. TW and NK supervised genome sequencing at the DOE JGI. SS wrote the manuscript with input from all co-authors.

## Conflict of Interest Statement

The authors declare that the research was conducted in the absence of any commercial or financial relationships that could be construed as a potential conflict of interest.

## Footnotes

1 http://www.dsmz.de/microorganisms/medium/pdf/DSMZ_Medium141.pdf

2 http://www.dsmz.de/microorganisms/medium/pdf/DSMZ_Medium320.pdf

3 https://www.sigmaaldrich.com/

4 www.bdbiosciences.com/

5 https://www.dsmz.de/catalogues/catalogue-microorganisms.html

6 https://blast.ncbi.nlm.nih.gov/Blast.cgi

7 https://www.dsmz.de/microorganisms/medium/pdf/DSMZ_Medium641.pdf

8 http://gtdb.ecogenomic.org/

9 http://gtdb.ecogenomic.org/
