# Supplementary material for: Sulfate-Reducing Bacteria That Produce Exopolymers Thrive in the Calcifying Zone of a Hypersaline Cyanobacterial Mat
Source: Front Microbiol. 2019 Apr 24;10:862. doi: 10.3389/fmicb.2019.00862 (PMC6491731; doi:10.3389/fmicb.2019.00862)
Supplement: Supplementary file 1 [file Data_Sheet_1.docx]

**Supplementary Material**

**Sulfate-Reducing Bacteria That Produce Exopolymers Thrive in the Calcifying Zone of a Hypersaline Cyanobacterial Mat**

Stefan Spring, Dimitry Y. Sorokin, Susanne Verbarg, Manfred Rohde, Tanja Woyke, Nikos C. Kyrpides

**Contents Page**

Supplementary Tables S1 to S5 2 - 11

Supplementary Figures S1 to S3 12 - 14

**TABLE S1.** Specifications of genomes used for the reconstruction of phylogenetic relationships within the *Desulfovibrionales*.

| Accession No. ^a^ | Strain Designation ^b^ | G+C (mol%) | Size (Mb) |
| --- | --- | --- | --- |
| JOMJ00000000 | L21-Syr-AB^T^ | 65.47 | 3.39 |
| JNJP00000000 | *Bilophila wadsworthia* ATCC 49260^T^ (DsrBD fusion) | 59.22 | 4.63 |
| CM001488 | *Desulfobacter postgatei* 2ac9^T^ | 47.17 | 3.97 |
| JAEX00000000 | *Desulfocurvus vexinensis* DSM 17965^T^ | 69.66 | 3.63 |
| CP001734, CP001735 | *Desulfohalobium retbaense* DSM 5692^T^ | 57.33 | 2.91 |
| CP001629 | *Desulfomicrobium baculatum* DSM 4028^T^ | 58.65 | 3.94 |
| ACJN00000000 | *Desulfonatronospira thiodismutans* ASO3-1^T^ | 51.33 | 3.97 |
| JMKT00000000 | *Desulfonatronovibrio hydrogenovorans* DSM 9292^T^ | 50.33 | 2.94 |
| JAFE00000000 | *Desulfonatronum lacustre* DSM 10312^T^ | 59.32 | 3.76 |
| FNIN00000000 | *Desulfonauticus submarinus* DSM 15269^T^ | 32.47 | 2.10 |
| BDFE00000000 | *Desulfoplanes formicivorans* Pf12B^T^ | 49.81 | 3.00 |
| JIAK00000000 | *Desulfovermiculus halophilus* DSM 18834^T^ | 56.81 | 3.24 |
| AULZ00000000 | *Desulfovibrio africanus* subsp. *africanus* DSM 2603^T^ | 61.10 | 4.41 |
| CP000112 | *Desulfovibrio alaskensis* G20 | 57.84 | 3.73 |
| JNJA00000000 | *Desulfovibrio alcoholivorans* DSM 5433^T^ | 64.70 | 5.13 |
| ATHI00000000 | *Desulfovibrio alkalitolerans* DSM 16529^T^ | 64.48 | 3.11 |
| AUMA00000000 | *Desulfovibrio aminophilus* DSM 12254^T^ | 66.16 | 3.42 |
| AUCX00000000 | *Desulfovibrio bastinii* DSM 16055^T^ | 43.06 | 3.91 |
| FUYA00000000 | *Desulfovibrio bizertenis* DSM 18304^T^ | 52.09 | 3.27 |
| BBCB00000000 | *“Desulfovibrio brasiliensis”* JCM 12178 | 59.65 | 3.56 |
| AUCY00000000 | *Desulfovibrio cuneatus* DSM 11391^T^ | 53.50 | 3.36 |
| ATUZ00000000 | *Desulfovibrio desulfuricans* DSM 642^T^ | 57.36 | 3.39 |
| CP014229 | *“Desulfovibrio fairfieldensis”* CCUG 45958 | 60.90 | 3.70 |
| FNGA00000000 | *Desulfovibrio ferrireducens* DSM 16995^T^ | 42.78 | 3.87 |
| JONL00000000 | *Desulfovibrio frigidus* DSM 17176^T^ | 42.75 | 4.19 |
| AECZ00000000 | *Desulfovibrio fructosivorans* JJ^T^ | 63.85 | 4.67 |
| CP006585, CP006586 | *Desulfovibrio gigas* DSM 1382^T^ | 63.63 | 3.79 |
| FUYC00000000 | *Desulfovibrio gracilis* DSM 16080^T^ | 58.43 | 3.18 |
| IMG 2571042916 | *Desulfovibrio halophilus* DSM 5663^T^ | 60.96 | 3.38 |
| FO203522, FO203523 | *Desulfovibrio hydrothermalis* DSM 14728^T^ | 45.09 | 3.66 |
| IMG 2574180452 | *Desulfovibrio idahonensis* DSM 15450^T^ | 64.99 | 3.57 |
| AUBP00000000 | *Desulfovibrio inopinatus* DSM 10711^T^ | 49.09 | 5.77 |
| FNBX00000000 | *Desulfovibrio legallii* KHC7 | 64.80 | 2.70 |
| FRDI00000000 | *Desulfovibrio litoralis* DSM 11393^T^ | 36.87 | 2.74 |
| ATVA00000000 | *Desulfovibrio longus* DSM 6739^T^ | 63.64 | 3.70 |
| AP010904, AP010905, AP010906 | *Desulfovibrio magneticus* RS-1^T^ | 62.67 | 5.32 |
| FZOC00000000 | *Desulfovibrio mexicanus* DSM 13116^T^ | 65.60 | 3.54 |
| AQXE00000000 | *Desulfovibrio oxyclinae* DSM 11498^T^ | 59.11 | 3.33 |
| IMG 2571042346 | *Desulfovibrio paquesii* DSM 16881^T^ | 62.98 | 4.21 |
| LT630450 | *Desulfovibrio piger* FI11049 | 64.20 | 2.81 |
| AUBQ00000000 | *Desulfovibrio putealis* DSM 16056^T^ | 62.79 | 4.71 |
| CP001649 | *Desulfovibrio salexigens* DSM 2638^T^ | 47.09 | 4.29 |
| DIXN00000000 | *Desulfovibrio* sp. MAG UBA6079 | 67.30 | 4.54 |
| ATHV00000000 | *Desulfovibrio* sp. X2 | 67.99 | 3.91 |
| AZAO00000000 | *Desulfovibrio termitidis* HI1^T^ | 66.07 | 4.26 |
| IMG 2574179706 | *Desulfovibrio vietnamensis* DSM 10520^T^ (*D. senezii* DSM 8436^T^) ^c^ | 61.39 | 3.99 |
| AE017285, AE017286 | *Desulfovibrio vulgaris* Hildenborough^T^ | 63.28 | 3.77 |
| AUDC00000000 | *Desulfovibrio zosterae* DSM 11974^T^ | 41.75 | 4.10 |
| AULY00000000 | *Halodesulfovibrio aestuarii* DSM 10141 | 45.02 | 3.41 |
| FSRG00000000 | *Halodesulfovibrio marinisediminis* DSM 17456^T^ | 44.95 | 3.71 |
| JXMS00000000 | *Halodesulfovibrio spirochaetisodalis* JC271^T^ | 46.19 | 3.61 |
| CP004029, CP004030, CP004031, CP004032 | *Lawsonia intracellularis* N343 | 33.08 | 1.72 |
| CP002431 | *Pseudodesulfovibrio aespoeensis* Aspo-2^T^ | 62.56 | 3.63 |
| LKAQ00000000 | *Pseudodesulfovibrio hydrargyri* BerOc1^T^ | 63.80 | 4.08 |
| CP014206 | *Pseudodesulfovibrio indicus* J2^T^ | 63.50 | 3.97 |
| FO203427 | *Pseudodesulfovibrio piezophilus* C1TLV30^T^ | 49.93 | 3.65 |

^a^ All accession numbers are from NCBI GenBank, except IMG numbers, which are from the JGI IMG/M database.

^b^ Superscript T denotes a type strain. Species names in quotation marks have been effectively published but not validly published under the rules of the International Code of Nomenclature of Bacteria.

^c^ It turned out that the determined sequences of the *Desulfovibrio senezii* dissimilatory sulfite-reductase genes obtained by cloning (JF830006) are 100% identical to the respective genes in the genome deposited under the name *Desulfovibrio vietnamensis* in the JGI IMG database, which is not possible due to the large phylogenetic distance of both species. Therefore, we conclude that the genomes of both species were mixed up at IMG and use in this study the designation *D. senezii* for the genome with the IMG ID 2574179706.

**TABLE S2.** Assembly statistics and sources of genomes representing a clade of sulfate-reducing bacteria containing the newly isolated strain L21-Syr-AB^T^.

| Organism | Unclassified species | Unclassified species | *D. alkalitolerans* | Metagenome | *D. africanus* subsp. *africanus* |
| --- | --- | --- | --- | --- | --- |
| Strain | L21-Syr-AB^T^ | X2 | DSM 16529^T^ | UBA6079 | DSM 2603^T^ |
| Source | hypersaline mat | estuarine sediment | biofilm growing in alkaline waters of a district heating system | oil sand tailings pond | well water |
| Assembly accession no. | GCA_ 000711295 | GCA_ 000422205 | GCA_ 000422245 | GCA_ 002428705 | GCA_ 000422545 |
| Total length (bp) | 3,390,909 | 3,912,295 | 3,202,328 | 4,541,696 | 4,402,525 |
| Ungapped length (bp) | 3,390,909 | 3,912,295 | 3,202,328 | 4,541,423 | 4,400,400 |
| Contig count | 5 | 66 | 32 | 78 | 44 |
| Contig N50 | 2,531,670 | 125,439 | 202,204 | 112,272 | 162,267 |
| Contig L50 | 1 | 10 | 6 | 15 | 9 |
| Scaffold count | - | - | - | 72 | 41 |

**TABLE S3.** Cellular fatty acid compositions of strain L21-Syr-AB^T^ and the related type strains *D. alkalitolerans* DSM 16259^T^ and *D. africanus* subsp. *africanus* DSM 2603^T^.

Values are percentages of total fatty acids. Major fatty acids (>5% of total amount) are given in bold; fatty acids that were detected only in trace amounts (<1.0% of the total amount) in all samples are not shown. Abbreviations: -, not detected; tr, trace amounts (<1.0% of the total amount); *c*, *cis* isomer; *i* and *ai* indicate *iso*- and *anteiso*-branched fatty acids, respectively.

| Fatty acid | L21-Syr-AB^T^ | DSM 16529^T^ | DSM 2603^T^ |
| --- | --- | --- | --- |
| *i*-C_14:0_ | 1.8 | tr | 1.0 |
| C_14:0_ | 2.6 | tr | 3.6 |
| *i*-C_15:1_ F ^a^ | - | - | 2.2 |
| *ai*-C_15:1_ A ^a^ | - | - | 1.2 |
| *i*-C_15:0_ | 7.1 | **30.4** | 3.0 |
| *ai*-C_15:0_ | **39.3** | **24.8** | **5.4** |
| *i*-C_16:1_ H ^a^ | - | - | **7.6** |
| *i*-C_16:0_ | **10.7** | 1.5 | 1.8 |
| C_16:1_ *c*9 | - | tr | **23.1** |
| C_16:1_ *c*11 | - | - | 1.6 |
| C_16:0_ | **21.5** | **7.4** | **8.6** |
| C_16:0_ 10 methyl | 1.9 | - | - |
| *i*-C_17:1_ *c*7 | - | **14.6** | **7.6** |
| *ai*-C_17:1_ *c*7 | - | **6.7** | **5.4** |
| *i*-C_17:0_ | 3.5 | **7.0** | tr |
| *ai*-C_17:0_ | **6.3** | 1.1 | tr |
| C_17:1_ *c*11 | - | - | 1.2 |
| C_18:1_ *c*11 | - | tr | **21.2** |
| C_18:0_ | 3.8 | 1.3 | tr |
| *i*-C_17:0_ 3OH | 1.0 | 2.8 | - |

^a^ Positions of double bonds were not determined.

**TABLE S4.** Classification, number and predicted activities of carbohydrate-active protein domains identified in the L21-Syr-AB^T^ genome.

| Carbohydrate-active enzymes or modules | No. of sequences ^a^ | Predicted activities |
| --- | --- | --- |
| *Carbohydrate-Binding Modules* |  |  |
| CBM48 | 2 | Glycogen-binding |
| *Carbohydrate Esterases* |  |  |
| CE4 | 1 | Polysaccharide deacetylase |
| CE11 | 1 | UDP-3-O-acyl-N-acetylglucosamine deacetylase |
| CE14 | 1 | GlcNAc-PI de-N-acetylase |
| *Glycoside Hydrolases* |  |  |
| GH3 | 1 | Beta-N-acetylhexosaminidase |
| GH13_3 | 1 | Alpha-1,4-glucan:maltose-1-phosphate maltosyltransferase |
| GH13_9 | 2 | 1,4-Alpha-glucan branching protein |
| GH13_10 | 2 | Malto-oligosyltrehalose trehalohydrolase |
| GH13_16 | 2 | Trehalose synthase |
| GH13_26 | 2 | Malto-oligosyltrehalose synthase |
| GH15 | 1 | Putative glucoamylase |
| GH23 | 2 | Lytic transglycosylase, Lytic murein transglycosylase C |
| GH57 | 2 | Alpha-amylase/alpha-mannosidase |
| GH65 | 1 | Beta-phosphoglucomutase |
| GH77 | 1 | 4-Alpha-glucanotransferase |
| GH102 | 1 | Membrane-bound lytic murein transglycosylase A |
| GH103 | 2 | Membrane-bound lytic murein transglycosylase B |
| *Glycosyltransferases* |  |  |
| GT1 | 1 | unknown |
| GT2 | 6 | Exopolysaccharide biosynthesis polyprenyl glycosylphosphotransferase, Undecaprenyl-phosphate mannosyltransferase |
| GT4 | 11 | GDP-mannose-dependent alpha-(1-6)-phosphatidylinositol monomannoside mannosyltransferase, Glycosyltransferase involved in cell wall biosynthesis, Alpha-D-QuiNAc alpha-1,3-galactosyltransferase, GDP-mannose-dependent alpha-mannosyltransferase, Trehalose synthase |
| GT5 | 1 | Glycogen synthase |
| GT9 | 6 | ADP-heptose:LPS heptosyltransferase, Lipopolysaccharide heptosyltransferase II |
| GT19 | 1 | Lipid-A-disaccharide synthase |
| GT20 | 1 | Trehalose 6-phosphate synthase |
| GT26 | 1 | Exopolysaccharide biosynthesis polyprenyl glycosylphosphotransferase |
| GT28 | 2 | UDP-2,4-diacetamido-2,4, 6-trideoxy-beta-L-altropyranose hydrolase, UDP-N-acetylglucosamine--N-acetylmuramyl-(pentapeptide) pyrophosphoryl-undecaprenol N-acetylglucosamine transferase |
| GT30 | 1 | 3-deoxy-D-manno-octulosonic-acid transferase |
| GT35 | 2 | Alpha-1,4 glucan phosphorylase |
| GT51 | 1 | Penicillin-binding protein |
| GT83 | 2 | Dolichyl-phosphate-mannose-mannosyltransferase, Phospholipid carrier-dependent glycosyltransferase |

^a^ Note that the total number is higher than the number of genes due to the occurrence of genes encoding more than one activity.

**TABLE S5.** Manually annotated genes with a potential function in the energy metabolism of strain L21-Syr-AB^T^. Assumed operons are highlighted with gray or blue shading. The IMG locus tags of the L21-Syr-AB^T^ genome are labelled with N911DRAFT_.

| Predicted protein | Gene Symbol | Amino Acids | IMG Locus Tag |
| --- | --- | --- | --- |
| Electron donor utilization |  |  |  |
| *Hydrogen* |  |  |  |
| Heterodisulfide reductase subunit A | *hdrA* | 669 | 3172 |
| Heterodisulfide reductase subunit C | *hdrC* | 169 | 3173 |
| Heterodisulfide reductase subunit B | *hdrB* | 305 | 3174 |
| F420-non-reducing [NiFe] hydrogenase iron-sulfur subunit | *mvhD* | 139 | 3175 |
| F420-non-reducing [NiFe] hydrogenase small subunit | *mvhG* | 325 | 3176 |
| F420-non-reducing [NiFe] hydrogenase large subunit | *mvhA* | 488 | 3177 |
| Hydrogenase maturation protease | *hybD* | 149 | 3178 |
| Membrane-bound Ech [NiFe] hydrogenase subunit F | *echF* | 193 | 0900 |
| Membrane-bound Ech [NiFe] hydrogenase subunit E | *echE* | 358 | 0901 |
| Membrane-bound Ech [NiFe] hydrogenase subunit D | *echD* | 120 | 0902 |
| Membrane-bound Ech [NiFe] hydrogenase subunit C | *echC* | 149 | 0903 |
| Membrane-bound Ech [NiFe] hydrogenase subunit B | *echB* | 282 | 0904 |
| Membrane-bound Ech [NiFe] hydrogenase subunit A | *echA* | 634 | 0905 |
| Periplasmic [NiFe] hydrogenase small subunit | *hynA* | 317 | 0964 |
| Periplasmic [NiFe] hydrogenase large subunit | *hynB* | 569 | 0965 |
| Periplasmic [NiFe] hydrogenase maturation factor | *hynC* | 178 | 0966 |
| Periplasmic [NiFe] hydrogenase assembly chaperone | *hynD* | 85 | 0967 |
| Type I cytochrome *c*_3_ | *cycA* | 132 | 3216 |
| *Formate* |  |  |  |
| Formate dehydrogenase maturation protein | *fdhE* | 292 | 1353 |
| Formate dehydrogenase assembly factor | *fdhD* | 231 | 1354 |
| Molybdopterin-guanine dinucleotide biosynthesis protein A | *mobA* | 200 | 1355 |
| Molybdenum cofactor biosynthesis enzyme | *moaA* | 330 | 1356 |
| Transposase |  | 296 | 1357 |
| Hypothetical protein |  | 67 | 1358 |
| Formate dehydrogenase major subunit | *fdhA* | 1005 | 1359 |
| Formate dehydrogenase iron-sulfur subunit | *fdhB* | 239 | 1360 |
| Formate dehydrogenase major subunit | *fdhA* | 1014 | 2430 |
| Formate dehydrogenase iron-sulfur subunit | *fdhB* | 248 | 2431 |
| Formate dehydrogenase maturation protein | *fdhE* | 316 | 2432 |
| Formate dehydrogenase assembly factor | *fdhD* | 253 | 2433 |
| *Ethanol* |  |  |  |
| Aldehyde:ferredoxin oxidoreductase | *aor* | 489 | 0054 |
| Rubrerythrin | *rbr* | 162 | 0055 |
| NAD(P)H-flavin reductase | *flxA* | 282 | 0056 |
| Fe-S oxidoreductase | *flxB* | 351 | 0057 |
| 4Fe-4S ferredoxin iron-sulfur binding domain-containing protein | *flxC* | 316 | 0058 |
| Methyl-viologen-reducing hydrogenase subunit D | *flxD* | 154 | 0059 |
| Heterodisulfide reductase subunit A | *hdrA* | 659 | 0060 |
| Heterodisulfide reductase subunit B | *hdrB* | 297 | 0061 |
| Heterodisulfide reductase subunit C | *hdrC* | 185 | 0062 |
| Alcohol dehydrogenase, class IV | *adh1* | 393 | 2511 |
| Two-component system response regulator, NtrC family |  | 451 | 2512 |
| PAS domain-containing protein |  | 401 | 2513 |
| Alcohol dehydrogenase, class IV | *adh2* | 379 | 2514 |
| *Pyruvate and Lactate* |  |  |  |
| L-Lactate utilization protein BA | *lutBA* | 727 | 1388 |
| L-Lactate utilization protein C | *lutC* | 210 | 1389 |
| Acetate kinase | *ackA* | 404 | 1390 |
| Phosphate acetyltransferase | *pta* | 704 | 1391 |
| D-Lactate dehydrogenase, iron-sulfur-binding domain | *glcF* | 426 | 1392 |
| D-Lactate dehydrogenase, FAD-binding domain | *glcD* | 461 | 1393 |
| Lactate permease | *lctP* | 570 | 1394 |
| Pyruvate:ferredoxin oxidoreductase | *por* | 1218 | 1395 |
| Two-component system response regulator, NtrC family |  | 477 | 1396 |
| PAS domain-containing protein |  | 848 | 1397 |
| FMN-dependent L-lactate dehydrogenase | *lldD* | 343 | 1398 |
| Sulfate reduction |  |  |  |
| Sulfate adenylyltransferase | *sat* | 426 | 0238 |
| Adenylylsulfate reductase beta subunit | *aprB* | 169 | 0239 |
| Adenylylsulfate reductase alpha subunit | *aprA* | 667 | 0240 |
| Quinone-modifying oxidoreductase, subunit A | *qmoA* | 411 | 0241 |
| Quinone-modifying oxidoreductase, subunit B | *qmoB* | 764 | 0242 |
| Quinone-modifying oxidoreductase, subunit C | *qmoC* | 415 | 0243 |
| Hypothetical protein |  | 228 | 0244 |
| Sulfite reductase-associated electron transfer protein DsrP | *dsrP* | 383 | 1528 |
| Sulfite reductase-associated electron transfer protein DsrO | *dsrO* | 257 | 1529 |
| Sulfite reductase-associated electron transfer protein DsrJ | *dsrJ* | 131 | 1530 |
| Sulfite reductase-associated electron transfer protein DsrK | *dsrK* | 535 | 1531 |
| Sulfite reductase-associated electron transfer protein DsrM | *dsrM* | 339 | 1532 |
| Hypothetical protein |  | 201 | 1533 |
| Dissimilatory sulfite reductase, subunit A | *dsrA* | 437 | 1562 |
| Dissimilatory sulfite reductase, subunit B | *dsrB* | 381 | 1563 |
| Dissimilatory sulfite reductase, subunit D | *dsrD* | 80 | 1564 |
| Cobyrinate a,c-diamide synthase | *cbiA* | 475 | 1565 |
| Hypothetical protein |  | 562 | 1566 |
| Sulfur relay protein DsrC | *dsrC* | 105 | 0568 |
| Membrane complexes involved in energy metabolism |  |  |  |
| *NADH-quinone oxidoreductase-like complex* |  |  |  |
| Subunit A | *nuoA* | 126 | 2747 |
| Subunit B | *nuoB* | 181 | 2748 |
| Subunit C | *nuoC* | 183 | 2749 |
| Subunit D | *nuoD* | 382 | 2750 |
| Subunit H | *nuoH* | 324 | 2751 |
| Subunit I | *nuoI* | 217 | 2752 |
| Subunit J | *nuoJ* | 169 | 2753 |
| Subunit K | *nuoK* | 102 | 2754 |
| Subunit L | *nuoL* | 494 | 2755 |
| Hypothetical protein |  | 87 | 2756 |
| Subunit N | *nuoN* | 596 | 2757 |
| Subunit M | *nuoM* | 522 | 2758 |
| Subunit N | *nuoN2* | 473 | 2759 |
| *Qrc complex* |  |  |  |
| Class III cytochrome *c* | *qrcA* | 186 | 2946 |
| Molybdopterin oxidoreductase | *qrcB* | 714 | 2947 |
| Molybdopterin oxidoreductase, iron-sulfur binding subunit | *qrcC* | 267 | 2948 |
| Molybdopterin oxidoreductase, membrane subunit | *qrcD* | 416 | 2949 |
| *Tmc complex* |  |  |  |
| Type II cytochrome *c*_3_ | *tmcA* | 132 | 2787 |
| Iron-sulfur oxidoreductase | *tmcB* | 448 | 2788 |
| Tmc subunit C | *tmcC* | 219 | 2789 |
| Tmc subunit D | *tmcD* | 420 | 2790 |
| *Rnf complex (ferrredoxin:NAD^+^ oxidoreductase)* |  |  |  |
| Class III cytochrome *c* |  | 251 | 0456 |
| Rnf electron transport complex subunit C | *rnfC* | 395 | 0457 |
| Rnf electron transport complex subunit D | *rnfD* | 312 | 0458 |
| Rnf electron transport complex subunit G | *rnfG* | 193 | 0459 |
| Rnf electron transport complex subunit E | *rnfE* | 242 | 0460 |
| Rnf electron transport complex subunit A | *rnfA* | 191 | 0461 |
| Rnf electron transport complex subunit B | *rnfB* | 710 | 0462 |
| Flavin transferase, ApbE-like |  | 324 | 0463 |
| *Ohc complex* |  |  |  |
| Iron-sulfur binding subunit | *ohcB1* | 335 | 1515 |
| Membran subunit | *ohcB2* | 159 | 1516 |
| Octaheme cytochrome *c* | *ohcA* | 547 | 1517 |
| Transmembrane cytochrome *b* | *ohcC* | 215 | 1518 |
| *F-type ATP synthase* |  |  |  |
| F_0_ subcomplex membrane subunit b’ | *atpF2* | 138 | 2300 |
| F_0_ subcomplex membrane subunit b | *atpF1* | 191 | 2301 |
| F_1_ subcomplex delta subunit | *atpH* | 183 | 2302 |
| F_1_ subcomplex alpha subunit | *atpA* | 502 | 2303 |
| F_1_ subcomplex gamma subunit | *atpG* | 298 | 2304 |
| F_1_ subcomplex beta subunit | *atpD* | 466 | 2305 |
| F_1_ subcomplex epsilon subunit | *atpC* | 142 | 2306 |
| F_1_ subcomplex beta subunit | *atpD2* | 461 | 3189 |
| F_1_ subcomplex epsilon subunit | *atpC2* | 127 | 3190 |
| F-type ATP Synthase membrane subunit I | *atpI* | 99 | 3191 |
| F-type ATP Synthase membrane subunit 2 | *atpR* | 96 | 3192 |
| F_0_ subcomplex membrane subunit a | *atpB* | 243 | 3193 |
| F_0_ subcomplex membrane subunit c | *atpE* | 93 | 3194 |
| F_0_ subcomplex membrane subunit b | *atpF3* | 282 | 3195 |
| F_1_ subcomplex alpha subunit | *atpA2* | 543 | 3196 |
| F_1_ subcomplex gamma subunit | *atpG2* | 300 | 3197 |
| *V-type proton ATPase* |  |  |  |
| V_1_ subcomplex subunit E | *ntpE* | 230 | 1707 |
| Hypothetical protein |  | 179 | 1708 |
| V_1_ subcomplex subunit A | *ntpA* | 577 | 1709 |
| V_1_ subcomplex subunit B | *ntpB* | 449 | 1710 |
| V_1_ subcomplex subunit D | *ntpD* | 201 | 1711 |
| V_0_ subcomplex subunit I | *ntpI* | 597 | 1712 |
| V_0_ subcomplex subunit K | *ntpK* | 160 | 1713 |

**FIGURE S1.** Polar lipid patterns of strain L21-Syr-AB^T^ and two related type strains after two-dimensional thin layer chromatography.


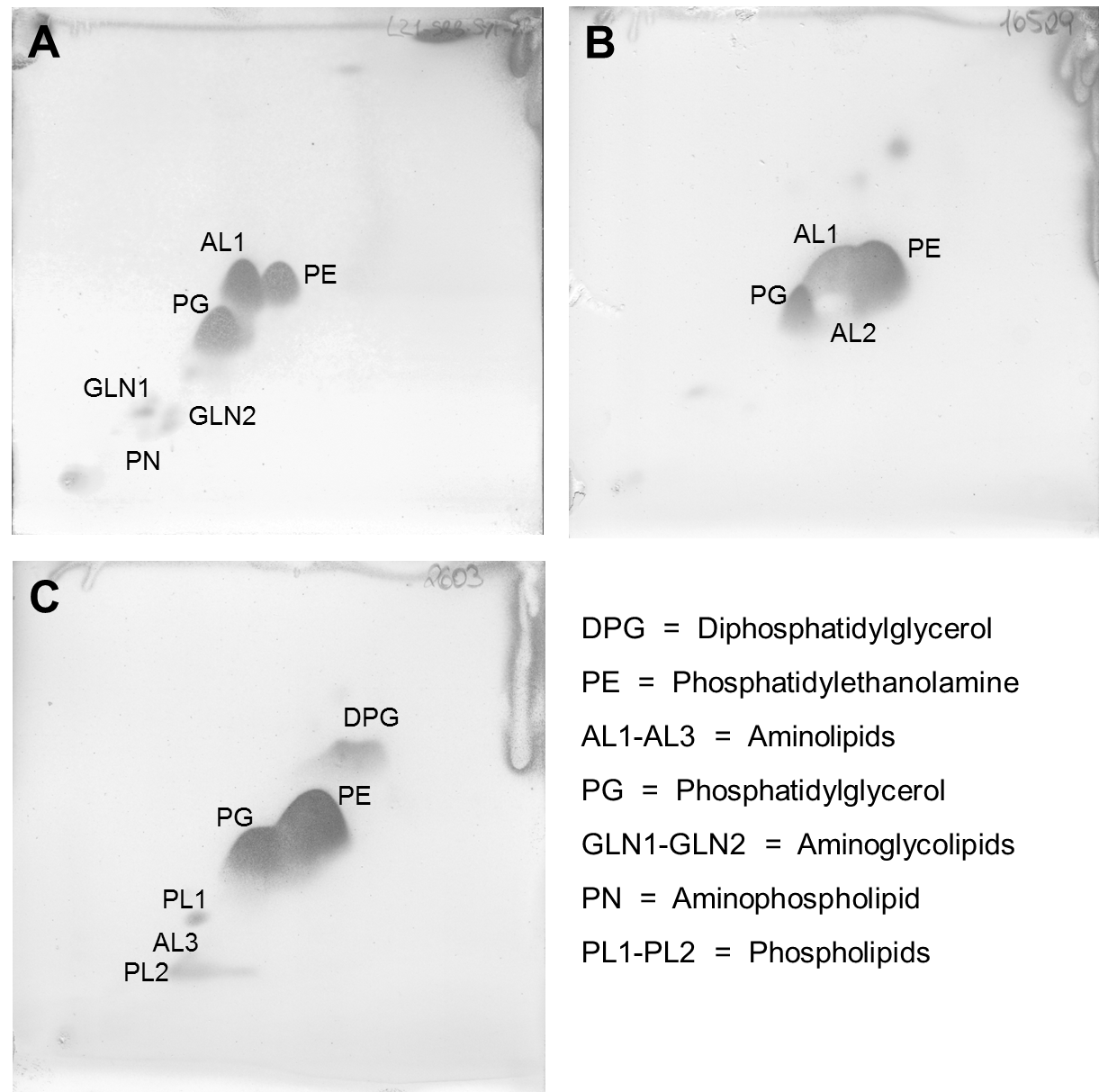


Chromatograms are shown for strain L21-Syr-AB^T^ **(A)**, *D. alkalitolerans* DSM 16529^T^ **(B)** and *D. africanus* subsp. *africanus* DSM 2603^T^ **(C)**. Staining of the chromatograms was done with molybdatophosphoric acid. Specific spray reagents were used to detect the functional head groups of the lipids.

**FIGURE S2.** Venn diagrams showing the shared gene content among strains of the L21-Syr-AB^T^ clade and the type species *D. desulfuricans*.


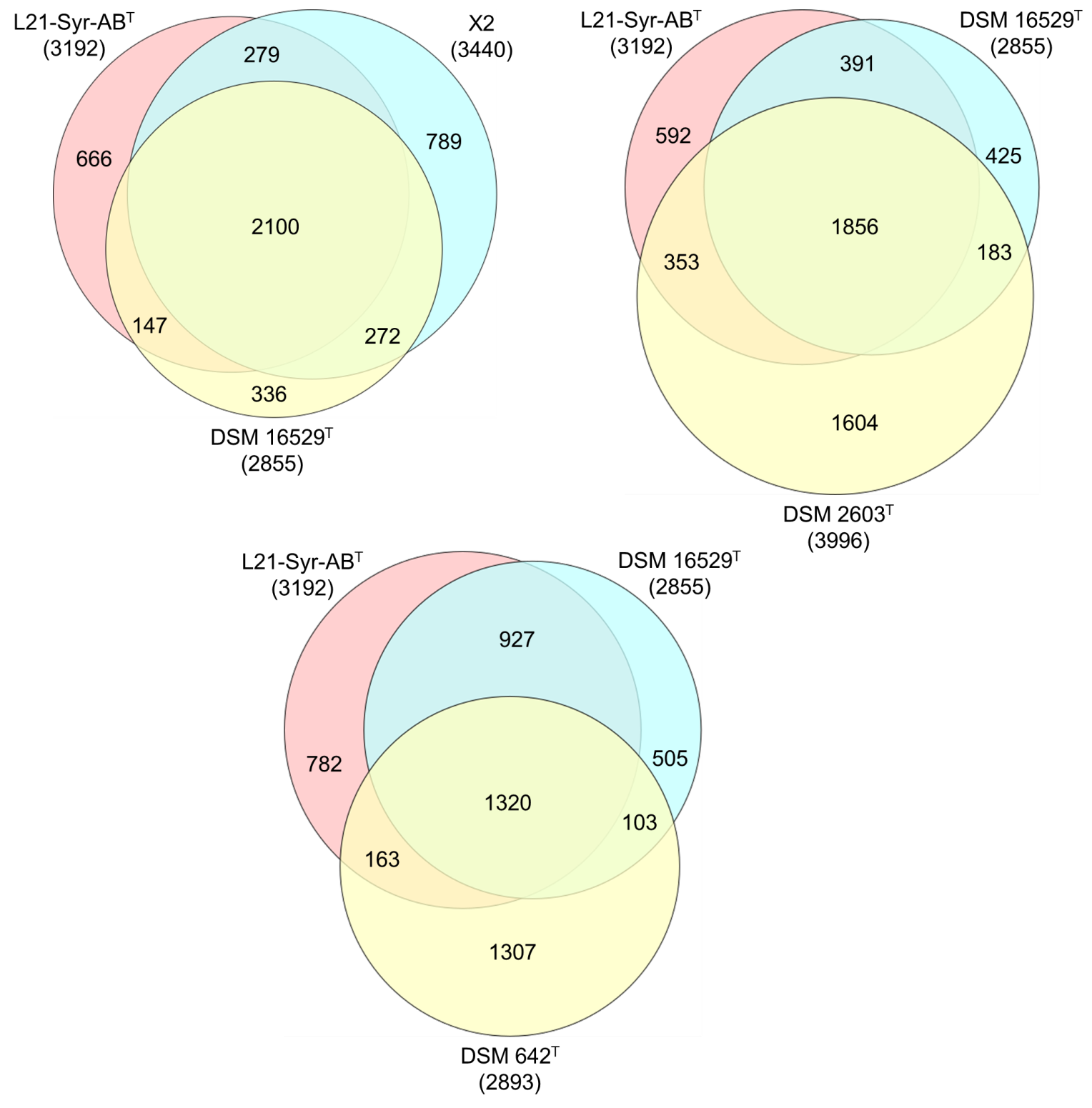


The number of overlapping protein genes is given inside the areas of the circles and the total number of protein genes used for each strain is shown in parentheses. Strain L21-Syr-AB^T^ was compared with *Desulfovibrio* sp. X2, *D. alkalitolerans* DSM 16529^T^, *D.* *africanus* subsp. *africanus* DSM 2603^T^ and *D.* *desulfuricans* DSM 642^T^. Diagrams were created using the Venn diagram plotter available from the Pacific Northwest National Laboratory Software Distribution Centre (<https://omics.pnl.gov/software/venn-diagram-plotter>).

**FIGURE S3.** Synteny plots of the L21-Syr-AB^T^ genome against related strains and the type strain of the type species *D. desulfuricans*.


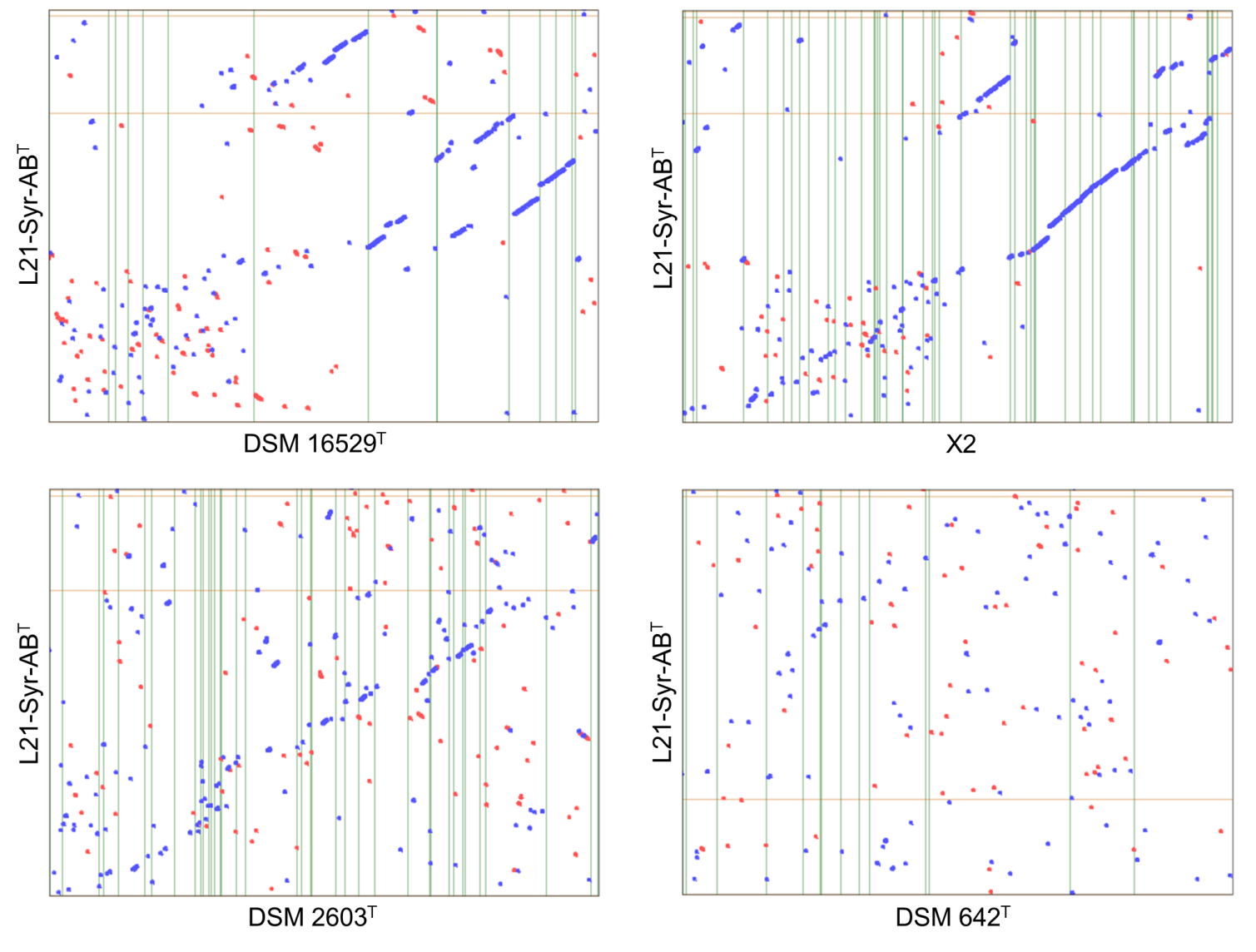


Strain designations given on the x-axis refer to *D. alkalitolerans* DSM 16529^T^, strain X2, *D. africanus* subsp. *africanus* DSM 2603^T^ and *D. desulfuricans* DSM 642^T^. The six frame amino acid translation of the DNA input sequences were used for comparing genomes using PROmer software. Blue dots represent regions of similarity found on parallel strands and red dots show regions of similarity found on anti-parallel strands.
